# Hepatitis C Virus: A Critical Appraisal of New Approaches to Therapy

**DOI:** 10.1155/2012/138302

**Published:** 2012-10-08

**Authors:** David R. Nelson, Donald M. Jensen, Mark S. Sulkowski, Greg Everson, Michael W. Fried, Stuart C. Gordon, Ira Jacobson, Nancy S. Reau, Kenneth Sherman, Nora Terrault, David Thomas

**Affiliations:** ^1^Clinical and Translational Science Institute, University of Florida, 1600 SW Archer Road, Room M440, Medical Science Building, Gainesville, FL 32610, USA; ^2^Center for Liver Diseases, University of Chicago Medical Center, Chicago, IL 60637, USA; ^3^Viral Hepatitis Center, The Johns Hopkins University, Baltimore, MD 21218, USA; ^4^University of Colorado Health Sciences Center, Aurora, CO 80262, USA; ^5^University of North Carolina at Chapel Hill, Chapel Hill, NC 27514, USA; ^6^Hepatology Section, Henry Ford Hospital, School of Medicine, Wayne State University, Detroit, MI 48201, USA; ^7^Division of Gastroenterology and Hepatology, Center for the Study of Hepatitis C, Weill Medical College of Cornell, New York, NY 10065, USA; ^8^Division of Digestive Diseases, College of Medicine, University of Cincinnati, Cincinnati, OH 45267, USA; ^9^Division of Gastroenterology, Viral Hepatitis Center, University of California San Francisco, San Francisco, CA 94143, USA; ^10^Infectious Diseases Viral Hepatitis Center, Johns Hopkins School of Medicine, Baltimore, MD 21287, USA

## Abstract

The HCV council 2011 convened 11 leading clinicians and researchers in hepatitis C virus from academic medical centers in the United States to provide a forum for the practical and comprehensive evaluation of current data regarding best practices for integrating new direct-acting antiviral agents into existing treatment paradigms. The council investigated 10 clinical practice statements related to HCV treatment that reflect key topical areas. Faculty members reviewed and discussed the data related to each statement, and voted on the nature of the evidence and their level of support for each statement. In this new era of DAAs, a comprehensive and critical analysis of the literature is needed to equip clinicians with the knowledge necessary to design, monitor, and modify treatment regimens in order to optimize patient outcomes.

## 1. Introduction

The 2011 approval of two HCV protease inhibitors, boceprevir and telaprevir, by the U.S. Food and Drug Administration (FDA) represents the most important advances in management of chronic HCV in nearly a decade. These protease inhibitors combined with pegylated interferon (PEG-IFN) and ribavirin (RBV) significantly improve the rate of sustained viral response (SVR) for all populations evaluated. Although the FDA has provided clear guidance in the labeling requirements for these agents, and new treatment guidelines have been developed [1], health care professionals will continue to depend on and need guidance from the most experienced specialists in the treatment of HCV in order to apply clinical trial data in the practice setting and maximize HCV outcomes.

The HCV Council 2011 convened 11 leading clinicians and researchers in hepatitis C virus (HCV) from academic medical centers in the United States to provide a forum for the practical and comprehensive evaluation of current data regarding best practices for integrating new direct-acting antiviral agents (DAA) into existing treatment paradigms. Ten clinical practice statements were developed (Table 1) that reflect key topical areas identified. Faculty members were responsible for reviewing the literature to support or reject these statements, which relate to current trends in HCV management. After review and discussion of the data, the Summit faculty voted on the nature of the evidence and their level of support for each statement (Table 2). In this new era of DAAs, a comprehensive and critical analysis of the literature is needed to equip clinicians with the knowledge necessary to design, monitor, and modify treatment regimens in order to optimize patient outcomes. The results of our detailed analysis with expert opinion are summarized below. (For full voting results from the Council, please see supplementary materials available at doi:10.1155/2012/138302).

## 2. Statement 1: PI/PEG-IFN/RBV Is the Standard of Care in All HCV Genotype 1 Treatment-Naïve Patients 

### 2.1. Rationale and Definition of Statement

With the approval of the protease inhibitors (PI) boceprevir and telaprevir, triple therapy (PI/PEG-IFN/RBV) is now available to treat patients with HCV. Statement 1 investigates whether triple therapy should be considered the new standard of care (SOC) in all genotype 1, treatment-naïve patients. Three phase 3, randomized clinical trials in treatment-naïve, HCV genotype 1 patients form the data set from which to assess the role of triple therapy with the HCV PIs, boceprevir, and telaprevir, in combination with PEG-IFN and RBV (Table 3). Both of these agents demonstrate significant improvements in SVR rates (SVR = 65%–75%) over SOC (i.e., PEG-IFN + RBV (PR)) (SVR = 38%–44%) [2–4]. 

### 2.2. Summary of Evidence

#### 2.2.1. Phase 3 Clinical Trial Subjects and Patient Selection

The success of the 2 HCV PIs makes these agents a first-line therapy for most HCV genotype 1 treatment-naïve subjects—but not all [2–4]. Is the lack of information about unstudied patient populations and undetermined drug-drug interactions (DDIs) sufficient to declare that triple combination therapy with a DAA + PEG-IFN/RBV is not the SOC for genotype 1 treatment-naïve HCV? Although some differences exist in the inclusion and exclusion criteria of the ADVANCE, ILLUMINATE, and SPRINT-2 trials, for the most part these were “healthy” HCV patients without significant comorbid disease [2–4]. If cirrhosis was present, it was well-compensated, and these patients represented a minority (<25%) of the enrolled subjects. HBV and HIV coinfection was an exclusion criteria for participation in these studies, as was organ transplantation, decompensated liver disease, and significant renal disease. Finally, to be eligible to enter these studies, patients had to be suitable candidates for PEG-IFN/RBV, since these agents remain an essential component of the triple-therapy success. 

The SVR rates in triple combination therapy were significantly better than SOC treatment across all genotype 1 studied patients, including those who are black, those who have high-baseline viral load or an unfavorable *IL28B* polymorphism, and those with advanced fibrosis or cirrhosis [2, 3]. Adverse events (AEs) related to the PI treatment infrequently led to treatment discontinuation, although they did necessitate the institution of specific countermeasures. Therefore, concern about potential AEs is unlikely to be a major reason not to initiate triple therapy. DDIs were not a major problem in the clinical trials, as patients were carefully selected to avoid potential exposure to therapeutic agents sharing a metabolic pathway with the PI. In clinical practice, these interactions have a potential to be more problematic, but this is rarely a reason not to select triple therapy. Treatment initiation decisions will require careful assessment of risks and benefits; however, these limitations are insufficient to preclude the recognition of triple-combination therapy as the new SOC. 

### 2.3. Discussion

In summary, the panel felt that triple-combination therapy with a PI + PEG-IFN/RBV will be the SOC for HCV genotype 1, treatment-naïve patients, in spite of some excluded subgroups and conditions. This combination is associated with significantly improved SVR rates compared to traditional SOC, but the therapy will be more complicated and will be associated with more potential AEs. Proper patient selection and a keen appreciation for the importance of stopping rules (Table 4), concomitant medication use, and side-effect management will be important for the translation of the positive phase 3 trial results into everyday practice. In addition, the potential added cost of triple therapy will become an important factor for drug-regimen selection in the setting of limited healthcare resources.

## 3. Statement 2: PI/PEG-IFN/RBV Is the Standard of Care in All HCV Genotype 1 Treatment-Experienced Patients

### 3.1. Rationale and Definition of Statement

As with Statement 1, this statement investigates the use of triple therapy as SOC in patients with HCV. The focus of this statement is on those patients who have failed previous treatment with PEG-IFN/RBV. Two major phase 3 studies evaluated the role of triple therapy with either boceprevir or telaprevir in combination with PEG-IFN/RBV therapy in genotype 1 treatment-experienced patients (Table 5) [7, 8]. In RESPOND-2, boceprevir plus PEG-IFN/RBV was investigated in prior partial responders (≥2log_10_ HCV RNA declined by week 12, but never achieved SVR) and relapsers [7]. A 4-week lead-in was included in each arm, which allowed some estimation of basal interferon (IFN) responsiveness and a response-guided arm provided information regarding required treatment duration [7]. In REALIZE, telaprevir plus PEG-IFN/RBV was studied in 3 patient populations: prior relapsers, partial responders, and null responders (i.e., those with <2log_10_ HCV RNA decline by week 12 of prior PEG-IFN/RBV therapy) [8]. One arm of this 3-armed study did include a 4-week lead-in of PEG-IFN/RBV, but there was no response-guided arm—all patients were treated for 48 weeks [8]. 

### 3.2. Summary of Evidence

In both trials, previous relapse patients fared exceedingly well with SVR rates ranging from 69% to 88% [7, 8]. In many ways, these patients responded similarly to treatment-naïve subjects. Prior partial responders did better on triple therapy than on PEG-IFN/RBV alone, with SVR rates of 52%–59% compared to 7%–15% for SOC [7, 8]. In fact, the greatest relative benefit to triple therapy occurred in these treatment-experienced groups. Prior null responders in the REALIZE trial, not surprisingly, had the poorest response rate (33%); however, this was still markedly better than SOC (5%). In RESPOND-2, subjects with a <1log_10_ HCV RNA response after 4 weeks of lead-in demonstrated a 34% SVR compared to 0% in the SOC group with a <1log_10_ response after 4 weeks.

The amount of baseline fibrosis was particularly detrimental in the prior null responders. The SVR rate in prior null responders with previously documented cirrhosis was 14% in the pooled telaprevir arms versus 10% in the SOC arm. This is the area of major concern in both prior null responders and patients with cirrhosis—the response rates are not particularly good. Patients with lesser degrees of fibrosis actually did fairly well. This was seen in both prior relapsers, where the response rates were robust across all histologic groups (86% to 84%), and in prior partial responders, who demonstrated a 72% SVR with minimal fibrosis and 56% with bridging fibrosis. However, the SVR rates did drop to 34% in patients with cirrhosis. Relatively few patients with cirrhosis and even fewer with cirrhosis and prior null response were included; thus, there are large confidence intervals around these data.

### 3.3. Discussion

In summary, the panel felt that the data clearly supported the use of triple therapy in treatment-experienced patients when compared to SOC. There are, however, some subgroups of patients with suboptimal response rates, including prior null responders and those with cirrhosis, yet these groups still performed substantially better on triple therapy than with SOC. Since the majority of prior nonresponders and all cirrhotics will require full 48 weeks of therapy, careful consideration to risk : benefit, cost, and future therapeutic options is recommended. 

## 4. Statement 3 

Response-guided therapy should be utilized in all:

- Treatment-naïve patients treated with PI/PEG-IFN/RBV regimens.
- Treatment-experienced patients treated with PI/PEG-IFN/RBV regimens.

### 4.1. Rationale and Definition of Statement

The attainment of superior rates of rapid virologic response (RVR) and SVR with the addition of PIs to PEG-IFN/RBV has been accompanied by the capacity to shorten the total duration of therapy in many patients, thereby sparing them unnecessarily protracted exposure to these medications and their side effects. The concept of truncating the duration of therapy in patients who meet criteria for rapid response is known as response-guided therapy (RGT), which was tested extensively in the phase 3 development programs of the PIs. This statement was designed to assess whether RGT is appropriate for the treatment of all patients with HCV. The use of RGT was evaluated for both treatment-naïve (Table 6) and treatment-experienced (Tables 7 and 8) patients.

### 4.2. Summary of Evidence

The results of the phase 3 trials of telaprevir and boceprevir have established a firm foundation for RGT in most treatment-naïve, genotype 1 infected patients (Table 9). In the ADVANCE trial, a regimen of telaprevir plus PEG-IFN/RBV for 12 weeks followed by 12 weeks of additional PEG-IFN/RBV (T12PR24) or a T8PR24 regimen was administered to patients with an extended rapid virologic response (eRVR) (i.e., those with an undetectable HCV RNA at weeks 4 and 12) [3]. If eRVR did not occur, patients received a T12PR48 regimen. Of the patients who received T12PR, which has become the approved regimen, 58% had an eRVR, and their SVR rate with 24 weeks of total therapy was 89% (92% in the reanalysis presented at the FDA Advisory Meeting) [3, 9]. This compares with an SVR rate of 54% in patients who did not have an eRVR, and who received a “tail” of treatments with 36 weeks of PEG-IFN/RBV. These results strongly suggest that 24 weeks of total therapy is sufficient for patients with an eRVR. Even more conclusively, in the ILLUMINATE study, a supportive, phase 3 trial, patients with an eRVR were randomized into a T12PR24 versus T12PR48 regimen. The results affirmed the solidity of the foundation for RGT by demonstrating SVR rates of 92% and 88% in eRVR patients with shorter and longer treatment durations, respectively [4]. However, since the data in cirrhotic patients in these 2 trials was quite limited, the package insert for telaprevir indicates that “treatment-naïve patients with cirrhosis who have undetectable HCV-RNA at weeks 4 and 12… may benefit from an additional 36 weeks of peginterferon alfa and ribavirin (48 weeks total) [5].” 

In SPRINT-2, the pivotal phase 3 study of boceprevir in treatment-naïve patients, there were 2 arms containing active boceprevir dosing [2]. In one of them, boceprevir-treated patients received 48 weeks of therapy (4-week lead-in with PEG-IFN/RBV, 44 weeks with boceprevir + PEG-IFN/RBV) regardless of rapidity of response. In contrast, in the RGT arm, patients who had undetectable HCV RNA from week 8 (i.e., 4 weeks of triple therapy) through week 24 stopped treatment at week 28, while those who had detectable HCV RNA at week 8 but became negative by week 24 received PEG-IFN/RBV lead-in for 4 weeks, boceprevir + PEG-IFN/RBV for 24 weeks, and then PEG-IFN/RBV for 20 weeks, for a total of 48 weeks of treatment. Overall, 44% of patients were eligible for the 28-week regimen. In Cohort 1 (i.e., nonblack patients), SVR rates were 67% with RGT and 68% with 48 weeks. Of patients who had undetectable HCV RNA at weeks 8–24, the SVR rates were 96% in the 48-week group and 97% in the 28-week (RGT) group. In Cohort 2 (i.e., black patients), SVR occurred in 42% of the RGT group and 53% of the 48-week group. However, in a modified intention-to-treat analysis including only patients who received at least one dose of boceprevir, the SVR rates were 47% versus 53%, because all five black patients who failed to receive boceprevir were in the RGT group. These data led to African American patients being considered eligible for RGT in the product label. Cirrhotics comprised <5% of the study population, and treatment for 48 weeks, including 44 weeks of boceprevir + PEG-IFN/RBV, is recommended for these patients. For patients with a more delayed response (i.e., HCV RNA detectable at week 8 but negative at week 24), boceprevir with PEG-IFN/RBV is recommended for 32 weeks after the 4-week lead-in phase, followed by 12 weeks of PEG-IFN/RBV for a total of 48 weeks [6]. This regimen represents 8 more weeks of boceprevir during the triple phase than was evaluated in the SPRINT-2 study, because post-hoc analysis of the “late responder patients” indicated SVR rates of 66% with RGT (PR4/boceprevir + PR24/PR20) and 75% with PR4/boceprevir + PR44 in the aggregated cohorts, which was thought to represent a potentially meaningful difference [6]. 

The phase 3 trial of telaprevir in treatment-experienced patients (the REALIZE study) did not evaluate RGT; patients in all categories were treated with telaprevir + PEG-IFN/RBV for 12 weeks followed by PEG-IFN/RBV for 48 weeks total [8]. The SVR rates were 86%, 57%, and 31% in relapsers, partial responders, and null responders, respectively. However, during the FDA approval process, several arguments led to 24 weeks being adopted in prior relapsers who are re-treated with telaprevir and PEG-IFN/RBV. First, the SVR rate in relapsers was even higher than in treatment-naïve patients. Second, given the fact that at least 25% of treatment-naïve patients are destined to relapse after a course of PEG-IFN/RBV therapy, it seems likely that if RGT were inferior to 48 weeks of therapy in these patients, such an effect should have resulted in inferiority for the 24-week arm in the ILLUMINATE study. Finally, relapsers with an RVR who were treated with 24 weeks in two phase 2 trials had SVR rates of more than 90% (49/52) [10, 11]. In contrast, a full 48 weeks treatment is recommended with telaprevir-based therapy in prior partial and null responders [5]. 

The RESPOND-2 study of boceprevir in treatment-experienced patients prospectively included relapsers and partial responders (i.e., those with >2log_10_ reduction in HCV RNA at treatment week 12) [7]. If HCV RNA was undetectable at 8 weeks, patients in the RGT arm received 4 weeks of lead-in therapy and 32 weeks of triple therapy (36 weeks total). Patients who had detectable HCV RNA at week 8 but undetectable at week 12 received an additional 12 weeks of PEG-IFN/RBV for a total of 48 weeks of therapy. The SVR rate for the RGT arm was 59%, and for the 48-week arm, the SVR rate was 66%. There was a difference in on-treatment response of 10%–15%, even at early time points (e.g., 8 weeks) when patients were receiving the same regimen. This difference and the subsequent difference in SVR between RGT and 48 weeks were driven largely by disparities in response among cirrhotic patients, which contributed to the recommendation that cirrhotic patients should not be considered eligible for RGT and should receive 44 weeks of boceprevir + PEG-IFN/RBV after the 4-week lead-in, whether treatment-naïve or treatment-experienced [6]. 

Prior null responders were not included prospectively in the RESPOND-2 study. Based on the SVR rates in patients with <1log_10_ HCV RNA reduction after 4 weeks of the PEG-IFN/RBV lead-in, these patients are included among those eligible for boceprevir-based therapy in the product label, but with the stipulation that they receive 48 weeks of treatment including 44 weeks of boceprevir + PEG-IFN/RBV [6]. This recommendation appears to be vindicated following approval by the presentation of data from null responders in the control arms of the pivotal trial who subsequently were given open-label boceprevir-containing therapy, and achieved an SVR rate of 38% [12].

### 4.3. Discussion

Based on these considerations, HCV Council panel members felt that RGT can be recommended for noncirrhotic, treatment-naïve patients who receive PIs, but that 48 weeks of total therapy should be strongly considered in cirrhotics, even if early response has occurred. Although black patients are included among those eligible for RGT, clinicians may choose to extend the duration of therapy on an individualized basis in patients within this group, at least until we have more data on this patient population.

For treatment-experienced patients, the panel considered RGT to be appropriate in prior relapsers, though some panelists indicated they might choose to extend therapy in cirrhotics who have relapsed previously. Although RGT is approved for prior partial responders who receive boceprevir, some clinicians may choose to complete a 48-week course of therapy with a PEG-IFN/RBV “tail” if the patient is tolerating treatment well. In prior null responders, no panelist felt that RGT was appropriate at this time. No recommendations about RGT can be made for patient populations in which PI-based therapy currently is unapproved, including HIV-coinfected patients, patients with more advanced cirrhosis, and those who are posttransplant. Results of further studies in these patient populations are pending.

## 5. Statement 4:**IL28B **Genotyping Should Be Performed in All Candidates for PI/PEG-IFN/RBV Therapy

### 5.1. Rationale and Definition of Statement

Several studies have demonstrated that a patient's likelihood of responding to PEG-IFN/RBV differs according to *IL28B* genotype, especially for genotype 1 HCV infection. *IL28B* genotype also correlates to a patient's response to PI + PEG-IFN/RBV, and this information may be useful to anticipate treatment duration and success. This statement investigates the usefulness of *IL28B *genotyping in the era of treatment with PIs.

### 5.2. Summary of Evidence

**Figure 1:**
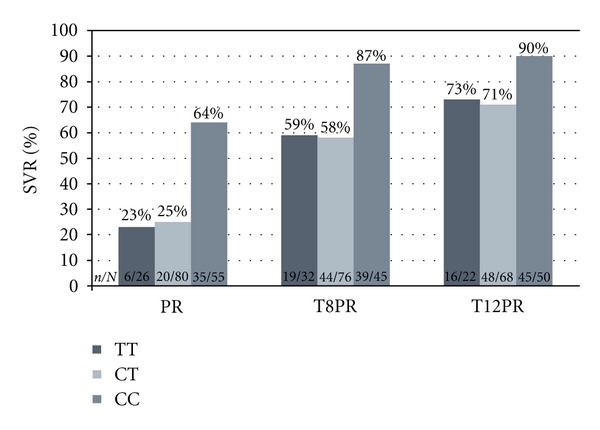
SVR rates in patients across All *IL28B *genotypes [16].

Evidence for *IL28B *genotyping in patients on triple therapy comes from one published randomized controlled trial and multiple look-back investigations of randomized, placebo-controlled trials. The likelihood that PEG-IFN/RBV treatment of HCV infection will result in an RVR or SVR depends on nucleotide sequences near *IL28B* [13]. Although several single-nucleotide polymorphisms are highly predictive of treatment response, none is more strongly associated than detection of the C or T allele at position rs12979860. Among Caucasians with chronic HCV genotype 1 infection treated with PEG-IFN/RBV, SVR is achieved in 69%, 33%, and 27% who have the CC, CT, and TT genotypes, respectively [14]. Among African Americans, SVR rates were 48%, 15%, and 13% for CC, CT, and TT genotypes, respectively. No other test is a stronger pretreatment predictor of response to PEG-IFN/RBV. Preliminary data indicate that *IL28B* genotype also predicts SVR with PI-based triple therapy [15–17]. Among Caucasian patients taking a telaprevir-based regimen, SVR was achieved by 84%–90%, 57%–71%, and 59%–73% of patients with CC, CT, and TT genotypes, respectively (Figure 1) [16]. In patients taking boceprevir-based regimen, SVR was achieved by 80%–82%, 65%–71%, and 55%–59% of patients with CC, CT, and TT genotypes, respectively [17]. Similar trends have been noted in treatment-experienced patients. Prior treatment response provides superior information on the likelihood of SVR with retreatment; thus, the test is less useful in that patient group. However, when prior treatment response information is not available, *IL28B* testing may be considered for its contribution to pretreatment decision-making.

In RGT algorithms, *IL28B* genotype also is predictive of treatment duration and the likelihood of receiving only 24–28 weeks of therapy for naïve patients. In Caucasian treatment-naïve patients randomized to telaprevir, eRVR was achieved in 64%–78%, 51%–57%, and 45%–50% of patients with CC, CT, and TT genotypes, respectively [16]. In the same analysis, the PEG-IFN/RBV placebo group had eRVR rates of 16%, 2%, and 0% in patients with CC, CT, and TT genotypes, respectively. In treatment-naïve patients randomized to boceprevir, the initial week 8 HCV RNA threshold was achieved in 82%–89% and 51%-52% of patients with CC and CT/TT genotypes, respectively [17]. 

### 5.3. Discussion

*IL28B* genotype is a strong predictor of response to PEG-IFN/RBV therapy, and also may provide important response information for patients and providers to make informed decisions regarding use of HCV PIs. Naïve patients with CC genotype may be more likely to initiate therapy knowing that there is a high likelihood of only needing 6 months of therapy. In the treatment-experienced patient, *IL28B* genotype may be most useful in patients whose prior therapy is not well-characterized. 

However, there are a number of unanswered questions regarding *IL28B* testing. The biologic basis of this striking association is unknown. In addition, the cost effectiveness of using *IL28B* testing to withhold HCV PI therapy from persons with favorable genotypes (e.g., CC) also is unknown. Moreover, it is not clear to what degree *IL28B* genotype test results predict response to HCV PI + PEG-IFN/RBV therapy for particular subsets of patients, such as African Americans, HIV-coinfected or posttransplant patients, and those with cirrhosis. Likewise, although further investigation is needed, there may be significantly less predictive value of *IL28B *testing as more potent HCV regimens emerge, as has been recently suggested by Aerssens and colleagues in the PILLAR trial [18]. 

## 6. Statement 5: Null Responders to Previous PEG-IFN/RBV with Minimal Liver Disease Should Not Be Treated with PI-Based Therapy 

### 6.1. Rationale and Definition of Statement

The basis of this statement is that prior null responders may have inadequate virologic response to retreatment with PI-based therapy rendering treatment futile, exposing patients to unnecessary risks, and favoring viral resistance. Patients with minimal disease are clinically stable, lack immediate need for treatment, and could benefit from waiting for future, more effective antiviral therapies. 

### 6.2. Summary of Evidence

Historically, null responders experience <1log_10_ drop in HCV RNA by week 4 and <2log_10_ drop by week 12 during prior treatment with PEG-IFN/RBV. Null responders have been included and evaluated in the telaprevir trials as those with a <2log_10_ drop by week 12. Poor response, a surrogate for null response, was defined retrospectively by the investigators in the boceprevir trials as those patients who achieved <1log_10_ drop in HCV RNA by week 4 of a lead-in treatment with PEG-IFN/RBV.

#### 6.2.1. Results in the Telaprevir Trial

The design of the REALIZE phase 3 trial utilized 48 weeks of PEG-IFN/RBV for all subjects, which included 144 null responders [8]. Rates of SVR in these null responders were 5% with PR48, 33% with 4 weeks PEG-IFN/RBV lead-in followed by T12PR44, and 29% with T12PR48. In the REALIZE trial, null responders in the lead-in T12PR44 arm who experienced ≥1log_10_ drop in HCV RNA during lead-in had an SVR rate of 54% (15/28) compared to an SVR of only 15% (6/41) in patients with <1log_10_ drop in HCV RNA [19]. The relationship of SVR to fibrosis stage also was analyzed in the REALIZE study for the combined telaprevir arms and compared to the PR48 control [20]. Rates of SVR were 41%, 39%, and 14% in the telaprevir arms compared to 6%, 0%, and 10% for PR48, according to METAVIR F0–F2, F3, and F4 stages, respectively. Null responders with minimal liver disease (SVR 41%) were the most likely to respond to PI-based therapy.

#### 6.2.2. Results in the Boceprevir Trial

In the Phase 3 RESPOND-2 trial, 259 relapsers, 144 partial responders, but no null responders were enrolled [7, 20]. All patients in this 3-arm trial received a lead-in of 4 weeks of PEG-IFN/RBV followed by either 44 weeks PEG-IFN/RBV, 32 weeks boceprevir + PEG-IFN/RBV (RGT), or 44 weeks boceprevir + PEG-IFN/RBV (boceprevir44). Patients in the RGT arm with negative HCV RNA at weeks 8 and 12 stopped all treatment at week 36. During the lead-in, 102 patients had <1log_10_ decline in HCV RNA, and were classified as “poor responders” to IFN. Rates of SVR in these poor responders were 0%, 33%, and 34% for SOC, RGT, and boceprevir 44 groups, respectively. This “poor responder” lead-in group has been proposed as a potential surrogate for a null population, but as pointed out previously, the lead-in response may not be indicative of a prior null response [19].

#### 6.2.3. Summary of the Results of PI-Based Treatment of Null or Poor Responders to Prior PEG-IFN/RBV

Overall, the likelihood of SVR when null or poor responders to PEG-IFN/RBV are retreated with PI-based triple therapy is 29% to 41%. Although, these rates of SVR are lower than those achievable in treatment-naïve patients (SVR~75%) or relapsers (SVR~80%), the 41% SVR of null responders with METAVIR F0-F2 is similar to the SVR rates that justified widespread use of PEG-IFN/RBV in the treatment of naïve patients with genotype 1 HCV.

### 6.3. Discussion

#### 6.3.1. Emerging Future Treatment Options

The dilemma for null responders with minimal liver disease is whether to initiate PI-based triple therapy now, or wait for new therapies to emerge. In the INFORM-1 study, 8 null responders (7 infected with HCV genotype 1a) were treated for 13 days with an inhibitor of HCV polymerase (RG-7128, mericitabine) plus an inhibitor of HCV NS3/4A protease (danoprevir), and achieved median HCV RNA decline of −4.9log_10_ IU/mL (−5.2 to −4.5) [21]. Four of 11 null responders (45%) achieved SVR12 after 24 weeks of dual therapy with an NS5A inhibitor (BMS-790052) plus protease inhibitor (BMS-650032) [22]. In the same report, 10 of 10 null responders (100%) achieved SVR12 after treatment with the same dual regimen plus PEG-IFN/RBV [22]. Additional studies of other combinations of DAAs currently are underway. Undoubtedly, null responders will have more effective treatment options in the future—but, when? Since no combination therapy has yet entered into a phase 3 clinical trial, these treatment options are still 3 to 5 years into the future. Further, there is no guarantee that any of the regimens currently under investigation will satisfy the requirements for both safety and efficacy.

#### 6.3.2. Uncertainty in Defining Minimal Liver Disease

Minimal liver disease is characterized by lack of clinical manifestations; normal values for bilirubin, INR, and albumin; normal or only slightly elevated ALT; a METAVIR fibrosis score of F0-F2. In a study of 66 patients with chronic HCV infection who had normal ALT and a mean METAVIR fibrosis score of 0.9 ± 0.8, fibrosis progression was estimated at 0.07 ± 0.07 stage/yr [23]. Given this rate of fibrosis progression, after 5 years, the mean stage would have increased by only 0.35. But, patients with normal ALT often develop abnormal ALT, and up to two-thirds have portal fibrosis and 10% have bridging fibrosis or even cirrhosis [24]. In addition, biopsy often underestimates the stage of fibrosis, especially in fragmented specimens [25]. Also, 15%–19% of patients with normal ALT, especially those over age of 65, may have moderate or high fibrosis progression rates [26]. In a biopsy study from 2 European centers ~50% of patients with METAVIR F0-F1 progressed 2 stages over the course of 5 years [27]. In the HALT-C trial 5.6% of patients with Ishak F2 (equivalent to METAVIR F1) experienced a clinical outcome over 6 years of followup [25]. Thus, a proportion of null responders with minimal liver disease could experience significant progression in fibrosis and even experience a negative clinical outcome while waiting the possible 3–5 years for the next generation of HCV therapies.

#### 6.3.3. Concluding Remarks

An SVR of 41% is sufficient for recommending treatment with triple therapy in prior PEG-IFN/RBV null responders, even those with minimal disease. The alternative to treatment now is to await future, more effective therapies, but these may be years away, and there is no guarantee that minimal liver disease will not progress during that time. Because of this and the ability to utilize early viral stop rules in those patients not responding to triple therapy, the panel recommended *consideration* of PI-based therapy for null responders with minimal liver disease.

## 7. Statement 6: Viral Resistance Testing Has No Clinical Utility in the Management of HCV Patients Receiving PI/PEG-IFN/RBV Therapy 

### 7.1. Rationale and Definition of Statement

While used in combination with PEG-IFN/RBV, PIs demonstrate high rates of SVR. However, treatment failures frequently result in the selection of a population of HCV variants that harbor unique mutations in their genome, which renders the variant less susceptible to the specific agent. Interestingly, prior to treatment with PIs, variants that carry potential resistance mutations can be detected in baseline samples of 5% to 7% of persons; however, the presence of detectable variants has not been strongly linked to treatment failure with combination therapy of a PI + PEG-IFN/RBV [5, 6]. Further, the clinical relevance of the selection of these resistant variants in persons who have failed therapy has not been defined. The role of genotypic testing for the presence of these resistance mutations before therapy or after unsuccessful therapy is unknown. This statement investigates the body of evidence surrounding the utility (or lack of utility) of viral resistance testing.

### 7.2. Summary of Evidence

The HCV genome is a 10,000-nucleotide positive-strand RNA that encodes for a large polyprotein with structural and functional components. The functional component includes a unique RNA-dependent RNA polymerase that the virus uses to replicate its positive-strand RNA through a negative-strand intermediate and back to multiple copies of the genomic RNA, which then can be packaged into new virions. This polymerase is highly error prone, resulting in the wrong nucleotide being inserted into replicating strands of the HCV genome. It is estimated that 1 error is made for every 10,000 nucleotides. In other words, every strand of new HCV RNA contains at least 1 replaced nucleotide, which may result in a change of the amino acid structure of the protein generated from that strand. This process, coupled with a very high rate of virus production (up to 12 trillion virions/day), ensures that every day the virus randomly creates every possible combination of nucleotide variability [28]. This mechanism (high-replication rate combined with an error-prone polymerase) allows the virus to adapt to the pressure of the immune system that is directed against unique epitopes in the viral coat (envelope). This adaptability permits the virus to escape eradication and establish chronic infection. Thus, in each individual patient, this mechanism results in the presence of a swarm of similar but still unique virus particles termed the “quasispecies.” The most common variant at a specific site in the population is termed the “wild type.” [29]. 

Since the PIs and other DAAs are highly targeted molecules designed to fit into the catalytic site of the viral coded functional enzymes, they act to perturb the viral population, leading to a survival advantage to naturally occurring variants that have mutations coding for minor changes in the amino acid, which renders the functional protein more or less able to bind a targeted drug at a specific site [28]. Variants with such changes are less susceptible to the DAA, and this confers a fitness advantage compared to other variants in the presence of the specific DAA. However, in the absence of selection pressure from the DAA, such variants may have relative decreased fitness compared to wild-type virus. In addition, resistant variants to a specific DAA remain susceptible to other antiviral agents, including PEG-IFN/RBV and DAAs which target different sites. 

The amino-acid changes conferring resistance to telaprevir and boceprevir are similar, with the identification of substitutions at positions V36M/A/L, T54A/S, R155K, and A156S/T conferring reduced susceptibility to the DAA [5, 6]. In an analysis of baseline specimens from the phase 3 trials of telaprevir and boceprevir, NS3-amino-acid substitutions were detected in 5%–7% of subjects using relatively insensitive population sequencing techniques [5, 6]. Interestingly, the presence of these PI resistance-associated substitutions at baseline did not preclude the patient achieving an SVR with combination therapy [5, 6, 30]; however, in the boceprevir trials, subjects with poor IFN responsiveness appeared to be less likely to achieve SVR when mutations were present at baseline [6]. Taken together, these observations suggest that PEG-IFN/RBV plays a critical role in preventing the selection of PI resistant variants, which leads to higher rates of SVR, despite the presence of these variants in the natural state. 

In the SPRINT-2 and RESPOND-2 trials, the majority of subjects who did not achieve SVR had one or more specific treatment-emergent NS3-amino-acid substitutions. Detection of these substitutions was most common in subjects with virologic breakthrough or incomplete viral suppression, but it also was detected in persons with on-treatment suppression followed by relapse after discontinuation of therapy [31]. Interestingly, differences in resistant variants were observed according to genotype 1 subtype (a or b). In the telaprevir studies, 69% of subtype 1a subjects who failed to achieve SVR had detectable resistant variants, primarily R155K/T and/or V36M. In contrast, 45% of subtype 1b subjects who failed to achieve SVR had detectable resistant variants, primarily V36A/L, T54A/S, or A156S/T [5]. Similar patterns according to subtype also were observed in the boceprevir trials. In both study programs, subjects with detectable PI-resistant variants at the end of treatment were followed for ~3 years with serial analysis of the viral population by population sequencing. In the telaprevir studies, at 36 months after removal of the selection pressure of the PI, V36M, T54A/S, and A156N/S/T variants had fallen below the level of detection, and 3% of the R155K variants still had detectable variants [5]. Due to the insensitivity of population-sequencing techniques, these data suggest but do not prove that the viral subpopulations harboring resistant variants declined to pretreatment levels. Of note, the analysis of PI resistance-associated variants was conducted retrospectively, and there are no data on the utility of real-time genotype testing for the presence of resistant variants pre-, during, or posttreatment. Similarly, there are no data on the retreatment of patients who failed to achieve SVR following treatment with a PI + PEG-IFN/RBV. 

### 7.3. Discussion

Commercial viral resistance testing has the ability to pick highly targeted sites and report on the nature of the amino acid present at that site. In July 2011, the first commercial PI drug resistance assay was made available in some countries [32]. This assay is sensitive to detect mixtures of wild-type and drug-resistant viruses when present at levels that represent >10% of the total virus populations and provides an interpretation of the significance of the variants detected for each PI (e.g., sensitive, resistant, and resistance possible). For example, if variants harboring the R155K variant are detected, the virus is relatively resistant to the HCV PI. However, the clinical significance of such testing prior to treatment is, at best, uncertain. As discussed previously, up to 7% of the patients in clinical trials of boceprevir and telaprevir had baseline dominant resistance mutations that likely would have been discovered with commercial resistance-testing modalities. Importantly, there was no difference in the treatment outcomes of these patients—they achieved SVR at the same rates as those without preexisting mutations. This probably is due to the antiviral activity of PEG-IFN/RBV, whose action is nonspecific and not tied to the presence of the resistance mutation. In patients who are relatively IFN nonresponsive, there is an increased rate of development of viral breakthrough due to the emergence of resistance mutations. However, these mutations are always present at some level below the level of detection of such assays, and are being newly generated, so resistance testing at baseline would not help to identify them. Thus, baseline resistance testing has no utility prior to the use of telaprevir or boceprevir + PEG-IFN/RBV. 

Following unsuccessful treatment with a PI-based regimen, virtually all patients have resistance mutations that slowly become undetectable over time, presumably returning to the low prevalence they had at baseline. Since the majority of patients who fail therapy will have detectable variants early after stopping the PI, there is little reason to test for them early after treatment, because they are always present and not informative. For later resistance testing, one must determine if knowing about the presence of these resistant variants at >10% of the total population would preclude treatment with different regimens that might contain a drug in the same class. Presently, there are no data that address this issue in patients with HCV. However, the presence of such variants may be overcome by several strategies. First, the use of the PI at concentrations above the new higher inhibitory concentration may suppress the virus. Second, the use of nonspecific (e.g., PEG-IFN) or other classes of agents (e.g., polymerase or NS5A inhibitors) that suppress the virus by a a different mechanism may lower the resistant virus polulation level. Finally, the use of a more potent agent (i.e., one less sensitive to mutations at that site compared to another agent in the same class) also has the potential to overcome resistance. As an example of this, prior resistance in HBV due to lamivudine exposure could be overcome by other nucleoside and nucleotide analog polymerase inhibitors (e.g., tenofovir). Thus, key data likely will be derived from future studies of re-treatment of patients previously exposed to PIs, either by use of drugs in other classes, other drugs in the same class, or inclusion of drugs used in a failed regimen within a broader therapeutic treatment approach. However, at this time, other anti-HCV drug options are not available, which renders information obtained through resistance testing difficult to utilize, as the presence or absence of detectable variants is not likely to alter current management. Thus, it was agreed by those on the Council that viral resistance testing has no clinical utility in the current management of HCV patients receiving a PI + PEG-IFN/RBV. 

## 8. Statement 7: Response to Lead-in Therapy Should Not Influence the Decision to Initiate a PI-Based Regimen

### 8.1. Rationale and Definition of Statement

Two strategies have been approved for the treatment of chronic HCV with PIs in combination with PEG-IFN/RBV: (1) delayed initiation of boceprevir following 4 weeks of treatment with PEG-IFN/RBV (lead-in) and (2) simultaneous initiation of telaprevir with PEG-IFN/RBV. The rationale for the lead-in strategy was based on the hypothesis that lead-in could reduce emergence of drug-resistant mutations by decreasing viral load and allowing PEG-IFN and RBV to reach steady-state pharmacokinetics before adding the PI [33]. Although the SPRINT-1 study did not firmly support this hypothesis, the HCV RNA decline (IFN responsiveness) during the lead-in phase was highly predictive of the likelihood of SVR with triple therapy. Lead-in was assessed further in the phase 3 clinic trials of boceprevir (with no comparison to simultaneous start) and in one phase 3 trial of telaprevir (randomized, with comparison to simultaneous start). This statement investigates the utility of lead-in and whether it can help identify patients who may not benefit from the addition of the PI either because they are highly IFN responsive (RVR) or poorly IFN responsive (null response) during the lead-in phase. 

### 8.2. Summary of Evidence

Two randomized placebo controlled trials evaluated the role of lead-in and its implications toward prevention of PI resistance-associated variants (RAVs). SPRINT-1, a phase 2 trial, included a 24- and 48-week regimen of therapy both with and without lead-in. The lead-in groups were associated with a modestly lower rate of breakthrough than were the groups with no lead-in (4% compared with 9% (*P* = 0.057)). Lead-in also allowed the authors to examine PEG-IFN/RBV responsiveness and SVR, which shaped the design of the subsequent boceprevir trials. Those participants experiencing <1.5log_10_ reduction in viral load during lead-in achieved SVR less frequently and may benefit from longer duration of therapy, as higher SVR rates were seen when participants received boceprevir + PEG-IFN/RBV for 44 weeks (79%–100% compared to 44%–65% with 24 weeks) [33].

REALIZE, a phase 3, double-blind, placebo-controlled trial, also evaluated the role of a 4-week PEG-IFN/RBV lead-in prior to the addition of telaprevir in a population of treatment-experienced patients. Lead-in did not have a significant impact on SVR rates (36% without versus 34% with lead-in failed to achieve SVR), virologic failure (20% compared to 17% with lead-in), or relapse rates (10% in both arms) [8, 20]. Given the ability of lead-in to predict SVR, two subsequent randomized, placebo-controlled trials included a lead-in in all boceprevir-containing arms. SPRINT-2 involved treatment-naïve patients [2]. Again those with less interferon responsiveness (<1log_10 _IU/mL decline in viral load at the conclusion of lead-in) experienced SVR less frequently with triple therapy (29%–39% compared to 82% in nonblack cohorts) [2]. RESPOND-2 included treatment-experienced patients, although patients with prior null response (<2log_10_ decline at treatment week 12) were excluded [7]. As was seen in SPRINT-2, response to lead-in was a strong predictor for SVR (73%–79% SVR with at least a 1log_10_ decline in viral load compared to 33%–34%). This was even stronger than historical response to therapy [7]. Samples from patients who failed to achieve SVR from both studies were sequenced for RAVs. Interferon responsiveness was predictive for the development of RAVs with a twofold higher incidence in those with poor interferon response (<1log_10_ viral load decrease with lead-in) [34].

Two double-blind, randomized, phase 2 trials involving either treatment-naïve patients (SILEN-C1) or patients with prior nonresponse to PEG-IFN/RBV (SILEN-C2) investigated a 3-day PEG-IFN/RBV lead-in prior to the addition of BI201335, a potent HCV NS3/4A protease inhibitor. Surprisingly, both studies found that the 3-day lead-in was associated with decreased viral response [35, 36].

### 8.3. Discussion

Although lead-in was conceptualized to decrease the emergence of RAVs, the data to support this hypothesis are limited. However, the data support that there is a potential use for lead-in to provide valuable information regarding current interferon responsiveness. First, as shown in the above studies, lead-in strongly predicts rates of SVR. In persons who achieve >1log_10_ reduction in HCV, this reinforces that the patient has IFN responsiveness, which predicts a high probability of SVR. Further, a minority of patients who achieve undetectable HCV RNA at the end of lead-in (RVR) have a high probability of achieving SVR with PEG-IFN/RBV alone. Some experts suggest that persons with an RVR may opt to avoid the addition of a DAA altogether and be treated with PEG-IFN/RBV; however, others argue that patients with an RVR after lead-in qualify for shortened therapy and would benefit from the addition of the DAA by limiting the overall duration of therapy and minimizing viral relapse. 

The outcome from lead-in also helps identify those at higher risk for treatment failure and the emergence of resistance. However, the reciprocal relationship (i.e., the ability of lead-in to predict treatment failure) is not accurate enough to discontinue HCV therapy prior to the introduction of a DAA. The design of the RESPOND-2 study included stopping therapy in all patients who still had replicating virus at treatment week 12 [7], while SPRINT-2 stopped patients at week 24 [2, 37]. In SPRINT-2 only 8%-9% of nonblack patients and 15%–17% of black patients stopped therapy at week 24, yet 30%–60% did not attain SVR, and resistance occurred in 40%–52% of patients with poor response to lead-in, confirming that a better stopping rule will be important. Indeed, the labeled recommendation is to discontinue all therapy in patients treated with boceprevir + PEG-IFN/RBV in whom the HCV RNA level is ≥100 IU/mL at treatment week 12 [6]. While informative, triaging patients based on lead-in also is inadequate. Importantly, patients with poor response to IFN (i.e., those who failed to achieve a 1log_10_ decline after 4 weeks of PEG-IFN/RBV) still attained SVR 25%–39% of the time compared to ~5% in the control group. Thus, restricting DAA access to only those patients who are IFN responsive (i.e., those with >1log_10_ decline after lead-in) would deny approximately one-third of patients the potential of obtaining an SVR. As such, lead-in data have limited utility in determining which patients should be treated with PIs. Although lead-in identifies a group with increased risk for resistance, it does so with limited accuracy to predict treatment failure. These data have not been utilized to modulate therapy, and thus one could argue that the lead-in is not necessary. 

However, there may be populations in which the risk of resistance is high, and the expected outcome even with triple therapy is low. In the REALIZE study, subjects with prior null response frequently (59%) experienced minimal viral decline with PEG-IFN/RBV lead-in and achieved SVR only 15% of the time [19]. This may be a population in which addition of a PI could be avoided to circumvent side effects, increased costs, and viral resistance, especially if the patients have minimal fibrosis and could delay treatment until more effective agents become available. 

Lead-in also offers insight into the patient's ability to tolerate and adhere to therapy. This helps identify subjects who are unable to comply with the increased complexity or side effect profile of a DAA. In other words, if the patient is unable to adhere to and/or tolerate 4 weeks of PEG-IFN/RBV, the likelihood of success with the addition of a PI can be expected to be vanishingly low. Additionally, the risk for PEG-IFN and RBV-induced anemia also can be predicted by hemoglobin (Hgb) decline at treatment week 2 [38]. This information may be useful to help formulate a strategy to manage anemia prior to the introduction of a DAA, which likely will exacerbate the decline in Hgb. Finally, despite the potential beneficial information regarding tolerability and interferon responsiveness, the application of clinical management decisions based on the results after lead-in may be difficult to implement in clinical practice due to the long (~7–10-day) turn-around time for HCV RNA testing in most settings. Thus, if the information gleaned through lead-in is intended to impact clinical management, the introduction of a third agent should be delayed until treatment week 5 or 6, after the results of the week 4 HCV polymerase chain reaction become available. 

Given the current information available, council members concluded that lead-in with PEG-IFN/RBV has limited impact on patient management and thus it should not influence the decision to initiate a PI, since both good and poor responders to PEG-IFN/RBV lead-in can benefit from the addition of the PI. However, the council also recognized that some clinicians may utilize lead-in as a measure of interferon responsiveness in some specific patient groups (e.g., prior null responders) to aid in the decision to start or withhold the PI and save the potential adverse events and cost associated with this therapy. 

## 9. Statement 8: Patients Treated with a Telaprevir-Based Regimen Who Develop a Severe Rash Should Be Switched to a Boceprevir-Based Regimen

### 9.1. Rationale and Definition of Statement

Telaprevir-associated rash was commonly observed in clinical trials, necessitating drug discontinuation in some patients [3, 39, 40]. The rationale behind this statement—switching from telaprevir to boceprevir in the event of severe rash—is in recognition of the substantial contribution of HCV PIs to achieving SVR in HCV genotype 1-infected patients and the need to exert continuous antiviral pressure, without drug discontinuation, to prevent the development of RAVs [41]. Attainment of a very rapid virologic response (HCV RNA negativity within the first 4 weeks of treatment) correlates with a higher likelihood of achieving SVR [42]. Failure to maintain potent HCV protease inhibition during the early course of therapy could diminish SVR and promote the emergence of protease-resistant variants.

### 9.2. Summary of Evidence

In the pivotal registration trials of telaprevir, rash/pruritus was reported in 56% of subjects taking telaprevir compared to 34% of those taking only PEG-IFN/RBV [5]. The rash is typically maculopapular and, in most cases, is associated with pruritus. Skin biopsy was performed in some patients, revealing spongiform dermatitis with a predominantly lymphocytic or eosinophilic perivascular infiltrate. Of note, these rashes were found to be clinically and histologically similar to those reported in patients on PEG-IFN/RBV. Severe telaprevir-associated rash necessitating discontinuation of telaprevir occurred in 6% of patients, with most rashes appearing early (usually during the first 16–20 days of treatment) [39, 40, 43]. Rash, as an event of special interest, was reported in 7% of the combined telaprevir groups versus <1% (i.e., 1 patient) for the combined PEG-IFN/RBV groups [39]. In addition, <1% of patients treated with telaprevir experienced more serious clinical manifestations of rash including Stevens Johnson Syndrome (SJS) and drug rash with eosinophilia and systemic symptoms syndrome (DRESS) [5]. Signs of SJS may include fever, target lesions, and mucosal erosions or ulcerations such as on the lips or conjunctivae, whereas the presenting signs of DRESS may include rash, fever, facial edema, and evidence of internal organ involvement, such as hepatitis or nephritis. The presence of systemic symptoms such as lymphadenopathy or hepatitis distinguishes a simple drug-related rash from DRESS, which has a reported mortality of 8%. Of note: rash severity does not necessarily correlate with the extent of internal organ damage. 

In the clinical trials, a rash-management plan was utilized, which guided the clinical management of telaprevir-associated rash. This plan has been largely incorporated into the FDA-approved prescribing information for telaprevir, with specific strategies recommended based on the severity of the rash [5]. Patients with mild-to-moderate rashes can be followed for progression of the rash (e.g., involvement of greater body surface area, mucosal surfaces) and/or the development of systemic symptoms (e.g., fever, edema). Treatment of mild-to-moderate rash with oral antihistamines and topical corticosteroids is recommended to provide symptomatic relief, but no data on the effectiveness of these measures are available. Treatment of rash with systemic corticosteroids is not recommended because the effectiveness of this is not known and there is concern for clinically significant DDIs with telaprevir [5]. Corticosteroids, such as prednisone and methylprednisolone, are CYP3A substrates. Since telaprevir is a potent CYP3A inhibitor, plasma concentrations of these corticosteroids can be increased significantly. If rash progresses and becomes severe or if systemic symptoms develop, discontinuation of telaprevir is required, while PEG-IFN/RBV can be continued. However, if no improvement is observed within 7 days of telaprevir discontinuation, interruption of PEG-IFN and/or RBV should be considered. After discontinuation of telaprevir, patients should be closely monitored until the rash has resolved. 

In the pivotal registration trials of boceprevir, boceprevir-related rash was reported, but these events were uncommon and not clinically severe [6, 31, 44]. However, while it is clear that telaprevir must not be restarted after it has been discontinued due to severe rash, there are no data on switching to boceprevir in such patients. Switching from telaprevir to boceprevir might be appealing, particularly in those who stop telaprevir relatively quickly after its initiation (e.g., prior to treatment week 8), since the likelihood of SVR may be reduced in such patients. 

### 9.3. Discussion

To date, anticonvulsants are the most frequent causative agent for DRESS (1 in 10,000 exposures), but the potential for serious cutaneous AEs may be higher for telaprevir than for previous FDA-approved drugs. However, patients and clinicians are reluctant to stop telaprevir because of its potent antiviral activity and evidence from the ADVANCE trial that indicates that 12 weeks of telaprevir therapy may lead to higher SVR rates than 8 weeks, particularly among patients with unfavorable *IL28B* genotypes (CT or TT) [3]. Interestingly, a similar debate appears in the literature and in clinical practice regarding whether to switch to an antiviral drug with a similar mechanism of action in the event of severe rash related to the treatment of HIV infection. Both nevirapine and efavirenz are nonnucleoside reverse transcriptase inhibitors (NNRTIs) of HIV replication that may cause rash, including DRESS. In a review of 239 patients with nevirapine rash challenged with efavirenz (after nevirapine dechallenge), 30 (12.6%) had rash recurrence [45]. Nevertheless, the World Health Organization recommends a substitution with efavirenz if the event is not life threatening [45]. In the case of telaprevir rash, however, a number of caveats may preclude or argue against a similar drug switch. Should the rash occur later during the course of therapy (i.e., after HCV RNA negativity is achieved), then PEG-IFN/RBV may be continued, and there may be limited potential advantage of exposure to an additional protease inhibitor. Furthermore, unlike the nevirapine dechallenge, the switch to boceprevir must be immediate in order to maintain antiviral pressure, so as not to sacrifice ongoing viral suppression. 

Prospective studies are needed to demonstrate the safety and efficacy of switching HCV PI drugs during the course of antiviral therapy. Nonetheless, an early (<4 weeks), severe telaprevir rash that necessitates drug discontinuation before the attainment of HCV RNA negativity could jeopardize the likelihood of SVR. Although some of the HCV Council members justified recommending such a switch under extenuating circumstances, the majority indicated that the evidence to support the practice of implementing a switch from telaprevir to boceprevir in patients who stop telaprevir for severe rash does not yet exist. 

## 10. Statement 9: In PI-Based HCV Treatment Regimens, Erythropoietin Should Be Used to Manage Anemia Prior to RBV Dose Reduction

### 10.1. Rationale and Definition of Statement

Due to suppression of hematopoiesis due to PEG-IFN and hemolysis due to RBV, anemia occurs frequently in patients undergoing HCV treatment and often leads to symptoms (e.g., fatigue), RBV dose reduction, and/or treatment discontinuation [46–48]. In some but not all studies of PEG-IFN/RBV therapy, RBV dose reduction was associated with lower SVR rates; and in some settings, erythropoiesis-stimulating agents (ESAs) have been used for the management of anemia to maintain RBV dosing and improve quality of life. In combination with PEG-IFN/RBV, telaprevir and boceprevir can cause an additional reduction in Hgb, leading to the higher incidence of anemia (defined as Hgb < 10 g/dL) compared to PEG-IFN/RBV alone (telaprevir: 45% versus 27%, boceprevir: 52% versus 32%) [39, 49]. Thus, anemia is a common and clinically important AE of telaprevir or boceprevir + PEG-IFN/RBV therapy. 

Due to differences in the duration of exposure to the HCV PIs (telaprevir: 12 weeks, boceprevir: 24 to 44 weeks), and the use of ESAs during clinical trials with boceprevir but not telaprevir, the management of anemia during HCV treatment is controversial. However, for both HCV PI regimens, there is concern that RBV dose reduction may compromise the antiviral efficacy of the regimen, leading to decreased likelihood of SVR. In addition, considerable uncertainty exists regarding the role of adjuvant ESAs, since the regulatory authorities have issued several advisories on potential AEs related to ESAs, including thrombovascular events and pure red blood cell aplasia [50–53]. This statement seeks to establish the evidence base for the use of ESAs prior to reducing RBV in patients being treated with a PI + PEG-IFN/RBV.

### 10.2. Summary of Evidence

Since the initial FDA approval of RBV for the treatment of HCV infection in 1998, RBV dose reduction has been recommended in patients with incident anemia, defined as Hgb < 10 g/dL. Subsequently, Hadziyannis and colleagues [54] found that higher SVR rates were observed in HCV genotype 1 infected patients treated with higher doses of RBV, and McHutchison and colleagues [55] reported that patients who can be maintained on >80% of their PEG-IFN/RBV dosage for the duration of treatment in the setting of a clinical trial had higher SVR rates. These observations led, in part, to studies of ESAs for the management of anemia in this patient population. In the study by Afdhal et al. [56], 88% of HCV-treated patients randomized to receive epoetin alfa maintained their RBV dose compared to 60% of those receiving sham injections (*P* < 0.001). In addition, patients treated with ESAs had an increase in Hgb level and improved symptoms. Several U. S. studies suggest that ESAs have been used in ~15% of patients treated with PEG-IFN/RBV. 

However, the 2009 American Association of the Study of Liver Diseases (AASLD) guidelines state that there are insufficient data to recommend the routine use of ESAs as a means to avoid or ameliorate RBV dose reductions in clinical practice [57]. This position has been supported by a retrospective analysis of 3023 HCV genotype 1 infected patients treated in the IDEAL study in which RBV dose reduction was not associated with lower SVR or higher relapse rates among patients treated with PEG-IFN/RBV [58]. Indeed, patients who developed anemia were more likely to achieve SVR compared to those with no anemia despite RBV dose reduction, leading to the hypothesis that the magnitude of Hgb decline is a pharmacodynamic marker of RBV exposure, correlating more closely with antiviral effect than the ingested RBV dose.

In the pivotal studies of telaprevir in combination with PEG-IFN/RBV, telaprevir led to an additional decline in Hgb level by 1.0 to 1.5 g/dL compared to PEG-IFN/RBV alone, a trend which appeared to reverse after discontinuation of telaprevir [39]. The incidence of anemia (defined as Hgb < 10 g/dL) was greater in patients treated with telaprevir, 36% compared to 17% in those treated with control [5, 39]. Interestingly, analysis by FDA reviewers found an exposure-response relationship for Hgb toxicity with both telaprevir and RBV concentrations; thus, subjects who developed anemia typically had higher exposure to both agents with a steeper exposure-response relationship observed with RBV [39]. In the telaprevir studies, anemia was managed with an RBV dose reduction to 600 mg/day (1-step); telaprevir dose reduction and/or use of ESAs were not permitted. Telaprevir discontinuation due to anemia occurred in 4% of patients, and RBV dose reduction, interruption, or discontinuation occurred in 32% of patients [5]. Blood transfusions were administered to 6% of patients (104 of 1797) [39]. The relationship of SVR, anemia, and RBV dose reduction has been retrospectively evaluated in the telaprevir studies of treatment-naïve patients (ADVANCE and ILLUMINATE); overall, the SVR was 76% (243 of 320 patients) in patients with RBV dose reduction and 72% in patients without dose reduction (408 of 565 patients) [59]. These data suggest that RBV dose reduction was not associated with decreased SVR rates in patients treated with telaprevir + PEG-IFN/RBV managed without adjuvant ESAs. Accordingly, the FDA-approved prescribing information for telaprevir recommends that anemia be managed with standard RBV dose reduction strategies, and if this approach is not adequate, telaprevir discontinuation should be considered [5]. The dose of telaprevir should not be reduced, nor should telaprevir be continued after RBV discontinuation.

Similarly, in the pivotal registration trials for boceprevir, anemia (defined as Hgb < 10 g/dL) also was more common in patients treated with boceprevir + PEG-IFN/RBV (52%) compared to the control group (32%) [49]. In addition, the FDA reviewers found a significant relationship between the incidence of anemia and the RBV concentration in the phase 3 trials in both the control- and boceprevir-treated patients. In these trials, the guidelines for the management of anemia permitted RBV dose reduction and/or use of ESAs in patients with Hgb < 10 g/dL. ESAs were provided at no cost to study participants. Overall, ESAs were used in 43% of the boceprevir-treated patients and 24% of those treated with PEG-IFN/RBV alone; RBV dose reduction also was more common in the boceprevir group (31%) compared to the control group (18%). Blood transfusions were administered to 4% of boceprevir-treated patients [49]. 

The relationships between SVR, RBV dose reduction, and anemia has been evaluated retrospectively in both treatment-naïve and treatment-experienced patients. Focusing on the treatment-naïve study (SPRINT-2), the SVR rate was higher (68%–78%) among patients who developed anemia compared to those who did not develop anemia (58%) [60]. While most anemic patients received adjuvant ESAs, the SVR rate among persons managed with RBV dose reduction alone (78%, 29 of 37 patients) or with RBV dose reduction plus ESAs (71%, 109 of 153 patients) was similar to that observed in anemic patients managed with ESA plus full-dose RBV (74%, 95 of 129 patients) [60]. Thromboembolic events were reported in clinical trials among subjects receiving the combination of boceprevir + PEG-IFN/RBV who were treated with ESAs; however, a causal relationship of the event and the ESA could not be established [49]. 

Despite confounding of data by the high rate of ESA use, these data suggest that RBV dose reduction was not associated with decreased SVR rates in patients treated with boceprevir + PEG-IFN/RBV. Accordingly, the FDA-approved prescribing information for boceprevir recommends that anemia be managed with standard RBV dose-reduction strategies and, if this approach is not adequate, RBV discontinuation should be considered [6]. The prescribing information for boceprevir describes the use of ESAs in the clinical trials without recommending their routine use. The dose of boceprevir should not be reduced, nor should boceprevir be continued after RBV discontinuation. 

### 10.3. Discussion

Anemia develops in approximately half of patients treated with HCV PI-based therapy, representing a significant challenge to the management of these patients. The issue at the center of the discussion is the debate surrounding the impact of RBV dose reduction on SVR. Data from 3023 patients treated in the IDEAL study failed to confirm a negative effect of RBV dose reduction on SVR with PEG-IFN/RBV; indeed, anemic subjects were more likely to achieve SVR than those who did not develop anemia, including those patients who reduced their RBV dose from 1000 or 1200 mg/day to 600 mg/day in one step [58]. This seemingly inconsistent response relationship likely can be explained by the finding that the incidence of anemia during RBV-based therapy is strongly associated with RBV exposure. The observation that higher SVR rates are observed in anemic patients compared to nonanemic patients may be related to higher RBV exposures. Thus, RBV dose reduction in such patients does not impair the likelihood of SVR. With this response-exposure relationship in mind, the data that the SVR rate is the same or higher among anemic patients treated with telaprevir or boceprevir plus PEG-IFN/RBV who undergo RBV dose reduction compared to nonanemic patients treated with full-dose RBV provide strong support for RBV dose reduction as the primary strategy for management of treatment-related anemia. 

On the other hand, the role of ESAs for the management of anemia during HCV treatment is controversial. In earlier randomized controlled trials, ESAs were associated with maintenance of RBV dose, increased Hgb, and improved quality of life in anemic patients treated with PEG-IFN/RBV. In addition, in the retrospective analysis of the IDEAL study, patients who rapidly developed anemia (within 8 weeks of starting PEG-IFN/RBV) achieved higher SVR rates if they received ESAs [58]. This effect was due to lower rates of treatment discontinuation in early anemic patients who received ESAs compared to those who did not receive ESAs; there was no apparent benefit of ESAs observed in those who developed anemia after treatment week 8. These data suggest that the primary role of ESAs may be to prevent treatment discontinuation in patients with severe symptoms attributed to anemia. 

However, the use of ESA also must be tempered by higher costs and the potential for additional AEs. In other patient populations, ESAs have been linked to increased risk of serious cardiovascular events, tumor progression, thrombosis, and death. Additionally, pure red blood cell aplasia due to neutralizing antibodies to native EPO has been reported during HCV treatment. The studies of ESAs during HCV treatment (including the boceprevir trials) are difficult to interpret from a safety perspective, as patients were not randomized, and ESA use was open-label. Of note, a randomized controlled trial of the management of anemia with RBV dose reduction alone or with RBV dose reduction plus ESAs is underway, and may shed additional light on the role of ESAs in this setting [61]. Nonetheless, ESAs are not approved by the FDA for the treatment of anemia in patients with HCV infection, and the use of ESAs in this patient population represents an off-label practice that is done at the discretion of treating physicians. Based on these considerations, members of the Council did not endorse the statement that erythropoietin should be used to manage anemia prior to RBV dose reduction in PI-based HCV treatment regimens. The Council firmly supported the use of RBV dose reduction as the primary strategy to manage incident anemia during treatment with telaprevir or boceprevir plus PEG-IFN/RBV.

## 11. Statement 10 

PI/PEG-IFN/RBV combinations are first-line therapy in:

- patients with HCV-HIV coinfection
- HCV-transplant populations.

### 11.1. Rationale and Definition of Statement

HCV-infected persons who are coinfected with HIV or who have undergone liver transplantation have a greater risk of liver-related complications due to more rapidly progressive HCV disease and have lower SVR rates following treatment than chronic HCV patients without these conditions [62–64]. Nonetheless, in both patient populations, successful HCV treatment with the achievement of SVR improves survival [65, 66]. Accordingly, there is considerable interest in using HCV PIs in combination with PEG-IFN/RBV for the treatment of HCV infection in these patients, despite added complexities of treatment. Enthusiasm for the use of HCV PI combination therapy is limited by several factors, including the lack of clinical trials establishing the safety and efficacy of these agents in these patient populations, the presence of DDIs with HCV PIs and antiretroviral and immunosuppressive agents, and the relatively high rate of AEs associated with PEG-IFN/RBV, including anemia. This statement seeks to evaluate the evidence for the use of PI + PEG-IFN/RBV as first-line therapy in these patient populations.

### 11.2. Summary of Evidence

Overall, the data to support the use of telaprevir and/or boceprevir in persons who are coinfected with HIV or have undergone liver transplantation are sparse. Key to effective treatment of patients with these conditions is DDI information. Limited DDI studies have been performed with more than 20 antiretroviral drugs as well as with the immunosuppressive agents cyclosporine, tacrolimus, and sirolimus. In terms of efficacy, there are small, phase 2, randomized studies of the HCV PIs in HIV coinfected patients, with only early virologic response data presented publically for one of these agents [67]. No studies have been conducted in liver transplant recipients. 

Further, there are limited data on the safety and efficacy of telaprevir and boceprevir in cirrhotic patients. The proportion of cirrhotics included in the pivotal registration trials of the PIs was low (≤10% in studies of treatment-naïve patients, and 20%–25% in studies with treatment-experienced patients) and only compensated cirrhotics without severe portal hypertension complications were included. 

#### 11.2.1. DDIs

In accordance with recommendations from the Department of Health and Human Services panel, most HIV-infected patients are receiving combination antiretroviral therapy with regimens that incorporate 6 mechanistic classes of drugs and may include pharmacologic boosting with the potent inhibitor of CYP3A, ritonavir [68]. Both telaprevir and boceprevir also interact with CYP3A as inhibitors and substrates, raising the potential for interactions with drugs that are metabolized through this pathway. Boceprevir is metabolized primarily by aldo-ketoreductase, but it is a strong inhibitor of and partially metabolized by CYP3A4/5 [6]. Similarly, telaprevir is an inhibitor and substrate of CYP3A. As such, drugs that are metabolized by CYP3A4 may have increased plasma concentrations, and drugs that induce this enzyme may lower telaprevir concentrations [5]. 

There are limited studies that assess interactions of the HCV PIs with antiretroviral agents. In healthy volunteer studies, when coadministered, boceprevir increased the concentrations of the NNRTI, efavirenz (an inducer of CYP3A), and the concentration of boceprevir was reduced. Similar interactions were observed with HIV PI, ritonavir, an inhibitor of CYP3A4 [69]. While interactions of boceprevir with other HIV PIs and other classes of antiretroviral agents are anticipated, these studies have not yet been presented. 

More extensive studies have been presented with telaprevir and antiretroviral agents. In studies of healthy volunteers, telaprevir was combined with the ritonavir-based HIV PIs, atazanavir, darunavir, fosamprenavir, and lopinavir [70]. The results were highly variable. Telaprevir led to significant reductions in the concentrations of the HIV PI with darunavir (AUC decreased by 40%) and fosamprenavir (AUC decreased by 47%), but there was less impact with lopinavir (AUC unchanged) and atazanavir (AUC decreased by 17%). Conversely, the HIV PIs also led to significant reductions in telaprevir concentrations (AUC decreased by 20% to 54%), with the smallest impact observed with atazanavir [70, 71]. Based on these studies, atazanavir boosted with ritonavir was permitted in the phase 2 study of telaprevir in HIV-infected patients; other HIV PIs were not allowed. With respect to the NNRTI, efavirenz, coadministration with this agent led to a 20% reduction in the AUC of telaprevir; this effect was offset by the administration of a higher dose of telaprevir, 1125 mg every 8 hours. This combination of higher dose telaprevir was studied in combination with efavirenz in the phase 2 study [67]. Finally, while studies have not yet been presented, the expectation is that no significant DDIs will be observed with the HIV integrase inhibitor, raltegravir, and the HCV PIs. 

Similar to the situation with antiretroviral agents in HCV-HIVcoinfected patients, HCV-infected transplant recipients must receive immunosuppressive agents. Calcineurin inhibitors (CNIs) and the drug sirolimus are metabolized by CYP34A, and drug interactions with both telaprevir and boceprevir are expected. DDI studies have been conducted with telaprevir and cyclosporine as well as tacrolimus [72]. In a phase 1 study of healthy volunteers, telaprevir coadministration with cyclosporine resulted in a 4.6-fold increase in dose-normalized exposure to cyclosporine, increasing the elimination half-life from 12 to 42 hours; whereas telaprevir coadministration with tacrolimus resulted in a 70-fold increase in dose-normalized exposure to tacrolimus, increasing the elimination half-life from 40.7 to 196 hours [72]. Thus, the pharmacokinetic effects of telaprevir are greater for tacrolimus than cyclosporine, and substantial reductions in CNI doses will be needed to prevent CNI toxicity. While DDI studies of boceprevir with cyclosporine and tacrolimus have not been performed, studies of boceprevir and midazolam, a well-recognized CYP34A inhibitor, indicate a 177% increase in *C*_max⁡⁡_ and 430% increase in AUC when boceprevir was coadministered with midazolam [6, 69]. These data strongly suggest that CNI dose reductions also will be necessary when boceprevir is used; whether the effect of boceprevir differs by type of CNI (as shown with telaprevir) is unknown. To date, no studies of DDIs with PIs and sirolimus have been presented.

#### 11.2.2. Safety and Efficacy in HCV-HIV Coinfected Patients

There is a preliminary report from a phase 2a study of 59 HCV-HIVcoinfected patients on antiretroviral therapy with tenofovir/emtricitabine or lamivudine with either efavirenz (*n* = 24) or ritonavir-boosted atazanavir (*n* = 22) or no antiretroviral therapy (*n* = 13) [67]. Patients were randomized to receive telaprevir plus PEG-IFN/RBV (12 weeks triple therapy followed by 36 weeks of PEG-IFN/RBV) or PEG-IFN/RBV for 48 weeks. The telaprevir dose was 1125 mg every 8 hours when the antiretroviral therapy regimen included efavirenz and 750 mg every 8 hours when the antiretroviral therapy regimen included atazanavir. At week 4 of treatment, 70% of telaprevir triple therapy patients had undetectable HCV RNA compared to 5% of PEG-IFN/RBV patients (71% (no antiretroviral therapy), 75% (efavirenz-based antiretroviral therapy), and 64% (atazanavir-based antiretroviral therapy)). Of 41 patients who reached 12 weeks of treatment, 49% of telaprevir-treated patients had eRVR. Discontinuations due to AEs occurred in 3% of the telaprevir-treated patients compared to none in the placebo group. Antiretroviral drug trough concentrations decreased by <20% when telaprevir was added, and there were no significant changes in control of HIV replication with no cases of HIV breakthrough observed. The median telaprevir trough, or lowest blood concentrations, was similar with and without antiretroviral therapy. There are no data on SVR in this population with telaprevir + PEG-IFN/RBV. With respect to boceprevir, a phase 2a study of boceprevir in combination with PEG-IFN/RBV is underway [73]. The phase 2a study investigating boceprevir in genotype 1 treatment-naïve coinfected patients compared 44 weeks of triple therapy with boceprevir in combination with PEG-IFN/RBV following the standard 4-week PEG-IFN/RBV lead-in phase (*n* = 64) to 48 weeks of PEG-IFN/RBV plus placebo (*n* = 34) [74]. To date, data from an interim, on-treatment analysis have been presented on 98 treated patients. At entry, all patients were required to have suppression of HIV replication on antiretroviral therapy, which included ritonavir-boosted HIV protease inhibitors, raltegravir, and nucleoside/nucleotide reverse transcriptase inhibitors, but not nonnucleoside reverse transcriptase inhibitors (e.g., efavirenz). At weeks 8, 12, and 24, 38%, 57%, and 71% of patients achieved an undetectable HCV RNA, respectively. AEs were common in both study arms: 7/34 (21%) patients in the control arm and 5/64 (8%) in the study arm experienced serious AEs, which lead to in discontinuation in 9% and 14% of patients, respectively. Primary AEs included pyrexia, anorexia, headache, dysgeusia, vomiting, and neutropenia. It is notable that anemia, the most common AE in the phase III HCV monoinfection studies, was similar across study arms and was not a primary reason for treatment discontinuation. There are no data on SVR rates in this population treated with boceprevir + PEG-IFN/RBV.

#### 11.2.3. Safety and Efficacy in Cirrhotic Patients Who Are Liver Transplant Candidates (Pretransplant)

There are data on the treatment of HCV-infected patients with compensated cirrhosis with telaprevir and boceprevir in combination with PEG-IFN/RBV, and these regimens have been approved by the FDA for the treatment of such patients [5, 6]. Among 82 treatment-naïve patients with cirrhosis treated with telaprevir + PEG-IFN/RBV enrolled in 2 studies, the rates of SVR were 62%–67% [3, 75]. Among 198 treatment-experienced patients with cirrhosis, SVR rates were 38%–48% compared with 8%–13% with PEG-IFN/RBV therapy [8, 40]. SVR rates were highest in relapsers and lowest in null responders. With telaprevir triple therapy, 84%, 34%, and 14% of relapsers, partial responders, and null responders achieved SVR, respectively, compared to 13%, 20%, and 10% treated with PEG-IFN/RBV alone [8, 20]. Among 15 treatment-naïve patients with cirrhosis and 76 with advanced fibrosis (METAVIR stage 3 and 4) treated with boceprevir + PEG-IFN/RBV, the rates of SVR were 67% [33] and 47% [2]. Among prior relapsers and partial responders with cirrhosis, SVR rates were 59% with boceprevir triple therapy for 48 weeks compared to 0% in PEG-IFN/RBV-treated patients [7]. SVR rates were significantly lower with response-guided boceprevir triple therapy than fixed-duration therapy: 35% versus 77% in treatment-experienced patients [7]. As such, the recommendation is that all patients with compensated cirrhosis (treatment-naïve and experienced) should be treated with boceprevir + PEG/IFN-RBV for 44 weeks following an initial lead-in phase of 4 weeks of PEG-IFN/RBV therapy [6].

Due to safety concerns, treatment of patients with decompensated cirrhosis with PEG-IFN/RBV is relatively contraindicated. Limited efficacy has been observed in the context of carefully conducted studies with, in some cases, gradual dose escalation of PEG-IFN/RBV, known as the LADR protocol (low accelerating dosage regimen). The prospect of the addition of boceprevir and/or telaprevir raises additional safety concerns as well as the potential for altered pharmacokinetics of these agents in the setting of hepatic impairment. After a single-dose of boceprevir in non-HCV infected subjects with decompensated cirrhosis, the mean AUC and *C*_max⁡⁡_ of the active diastereomer of boceprevir were significantly higher in patients with moderate (Child-Pugh-Turcott (CPT) score of 7–9), and severe (CPT score of 10–12) hepatic impairment relative to subjects with normal hepatic function (32% for moderate and 45% for severe) [6]. No dosage adjustment of boceprevir is recommended for patients with hepatic impairment. In contrast, the single-dose pharmacokinetic exposure in those with mild hepatic impairment (CPT score of 5-6) was similar to that of noncirrhotic patients [6].

For telaprevir, the steady-state exposure in HCV-negative subjects with mild (CPT class A, score 5-6) and moderate hepatic impairment (CPT class B) was reduced by 15% and 46% compared to healthy subjects [5]. Subjects with severe impairment (CPT class C) were not studied. Based on concerns about safety of triple therapy in patients with advanced cirrhosis and a lack of data for patients with hepatic impairment, boceprevir and telaprevir in combination with PEG-IFN/RBV are not recommended in patients with CPT score > 6. 

#### 11.2.4. Safety and Efficacy in Patients Who Are Liver Transplant Recipients (Posttransplant)

There are no data on the use of telaprevir or boceprevir in combination with PEG-IFN/RBV in this population. Studies will need to be conducted to evaluate the safety and efficacy of both PIs in patients after liver transplant.

### 11.3. Discussion

#### 11.3.1. HCV-HIV Coinfected Patients

Although the data are limited and incomplete, telaprevir or boceprevir may be recommended for use in combination with PEG-IFN/RBV for the treatment of chronic HCV infection in carefully selected HIV-infected patients not on antiretroviral therapies or on antiretroviral therapy regimens that are compatible with telaprevir or boceprevir [74]. Recognizing the substantial DDIs that may impact both antiretroviral and HCV PI concentrations, only those antiretroviral therapy regimens that have been evaluated in the clinical studies to date can be supported. Thus, tenofovir/emtricitabine plus either efavirenz (with telaprevir dosed at 1125 mg) or atazanavir/ritonavir are the antiretroviral therapy regimens recommended if HCV treatment with telaprevir is undertaken. In addition, an antiretroviral therapy regimen with a raltegravir backbone also is likely to be suitable, due to the absence of shared drug metabolism pathways. In contrast, boceprevir-based therapy may be used in coinfected patients taking ritonavir-boosted HIV PIs or raltegravir in combination with a nucleoside/nucleotide analogue backbone [74]; however, until additional data are available, boceprevir should not be used in patients taking efavirenz or other NNRTIs. Although SVR data are not available, the markedly higher RVR and complete EVR rates observed with telaprevir + PEG-IFN/RBV compared with PEG-IFN/RBV suggest that SVR rates also will be higher in this regimen, supporting the HCV Council's support of the statement in carefully selected patients with HIV/HCV coinfection.

#### 11.3.2. Cirrhotic Patients on the Liver Transplant Waiting List

In compensated cirrhotics, SVR rates with PI-based therapy are superior to those with PEG-IFN/RBV alone. Although the small number of patients studied to date limits accurate estimates of safety, tolerability, and efficacy, HCV PI-based therapy is the first-line therapy for this group. In contrast, HCV PIs plus PEG-IFN/RBV therapy are not approved or recommended for use in patients with decompensated cirrhosis. The risk of using PEG-IFN/RBV in this latter group is well established, and this therapy is generally restricted to use in select transplant centers. The addition of an HCV PI to PEG-IFN/RBV is associated with additional toxicity (e.g., anemia, rash); the potential for drug resistance in the setting of impending transplantation; and the expectation of low SVR rates. Of note, the use of lead-in therapy with PEG-IFN/RBV, regardless of the HCV PI used, may serve as a useful means of assessing the likelihood of response and establishing tolerability of PEG-IFN/RBV before exposing the patient to the PI (and its associated resistance). Additionally, clinicians need to be aware of the lack of information on telaprevir dosing in Child's B/C cirrhotics. The Council members did not support the routine first-line use of PIs in this patient population; however, this population is clearly of the highest medical need and consideration of treatment in a research setting was supported. 

#### 11.3.3. Liver Transplant Recipients

The risk oftelaprevir interactions and boceprevir interactions (although the latter has not been specifically studied) with CNIs and presumably sirolimus necessitates extreme caution in the use of these drugs in combination. Available data suggest a lesser effect of telaprevir on cyclosporine levels than on tacrolimus levels, so this may be a preferred CNI for patients undergoing PI triple therapy. Close monitoring of CNI levels is essential with the addition of a PI (i.e., with the risk of CNI toxicity) and removal of a PI (i.e., with the risk of acute rejection). The risks and benefits of undertaking treatment should be carefully considered pending additional data on the optimal dosing of PIs and immunosuppressive drugs. The Council members did not support the routine first-line use of PIs in this population; however, this population also is clearly of the highest medical need and consideration of treatment in a research setting was supported. 

## 12. Conclusion

The new era of DAAs is upon us and offers new hope for HCV-infected patients. The two oral protease inhibitors reviewed here, telaprevir and boceprevir, have completed phase 3 studies and have yielded some consistent early lessons for the protease class. For treatment-naïve, genotype 1 patients, higher cure rates and shorter duration of therapy can be expected, but these benefits are partially offset by new issues of resistance and increased AEs. SVR rates approaching 70%–75% now can be anticipated for treatment-naïve, genotype 1 patients, which should lead to increased treatment opportunities for many HCV populations. In addition, up to two-thirds of patients may be eligible for shorter duration therapy. In the treatment-experienced populations, relapse patients can expect to respond better than previous nonresponders (75%–86% versus 30%–60%, resp.) suggesting that IFN responsiveness is still an important consideration in selecting treatment candidates. Unfortunately, cirrhosis still plays a significant negative factor in response to PI-based triple therapy, especially in those with poor IFN responsiveness. In addition, new issues of viral resistance and increased AEs will increase the importance of close medical management and appropriate patient selection. 

Since *IL28B* polymorphism influences on-treatment virologic responses and has become the most powerful pretreatment predictor of SVR, it is anticipated that this host factor will continue to play a role with new PI-based, IFN-containing regimens. The approval of these new treatment regimens likely will expand the population of patients who will undergo antiviral therapy. However, it is important to understand that these drugs have only been studied extensively in a very homogenous population, which may not translate as well into more challenging populations (e.g., HIV co-infected patients, transplant patients, patients with renal failure). 

In conclusion, new DAA-based treatment regimens will offer higher SVR rates and new opportunities to many more of the treatment-naïve and treatment-experienced, genotype 1 populations. However, patient selection will be more important than ever given the complex nature of treatment decisions, monitoring, and side effect management.

## Supplementary Material

The purpose of this Council was to critically review the existing body of literature and analyze it in the context of contemporary practice and optimal therapeutic outcomes. Each panel member focused on the existing evidence pertaining to a specific controversy in the management of patients with HCV, and the Council as a whole evaluated and voted on the nature of the existing evidence for each statement. The Council members' evaluations of the evidence with level of support for each statement are shown in the supplemental materials.

## Figures and Tables

**Table 1:** HCV council statements for evaluation.

Workshop 1: Treatment strategies (statements 1–5)	
(1) PI/PEG-IFN/RBV is the standard of care in all HCV genotype 1 treatment-naïve patients.	
(2) PI/PEG-IFN/RBV is the standard of care in all HCV genotype 1 treatment-experienced patients.	
(3) Response-guided therapy should be utilized in all:	
(a) treatment-naïve patients treated with PI/PEG-IFN/RBV regimens.	
(b) treatment-experienced patients treated with PI/PEG-IFN/RBV regimens.	
(4) *IL28B *genotyping should be performed in all candidates for PI/PEG-IFN/RBV therapy.	
(5) Null responders to previous PEG-IFN/RBV with minimal liver disease should not be treated with PI-based therapy.	

Workshop 2: Treatment challenges (statements 6–10)	
(6) Viral resistance testing has no clinical utility in the management of HCV patients receiving PI/PEG-IFN/RBV therapy.	
(7) Response to lead-in therapy should not influence the decision to initiate a PI-based regimen.	
(8) Patients treated with a telaprevir-based regimen who develop a severe rash should be switched to a boceprevir-based regimen.	
(9) In PI-based HCV treatment regimens, erythropoietin should be used to manage anemia prior to RBV dose reduction.	
(10) PI/PEG-IFN/RBV combinations are first line therapy in:	
(a) patients with HCV-HIV coinfection.	
(b) HCV-transplant populations.	

**Table 2:** Council voting schemes.

Category	Nature of evidence
I	Evidence obtained from at least 1 well-designed, randomized, controlled trial
II	Evidence obtained from well-designed cohort or case-control studies
III	Evidence obtained from case series, case reports, or flawed clinical trials
IV	Opinions of respected authorities based on clinical experience, descriptive studies, or reports of expert committees
V	Insufficient evidence to form an opinion

	Level of support for each statement

1	Accept completely
2	Accept with some reservations
3	Accept with major reservations
4	Reject with reservations
5	Reject completely

**Table 3:** Responses in treatmsent-naïve patients.

	Boceprevir (BOC)	Telaprevir (T)	
	naïve	naïve	
Overall rates, % (*n*/*N*)	SPRINT-2 [2] BOC RGT + BOC/PR48 (ITT)	ADVANCE [3] T12/PR24/48 + T8/PR24/48	ILLUMINATE [4] T12/PR24 + T12/PR48 (ITT)

SVR	65 (475/734)	72 (521/727)	72 (388/540)
Relapse	9 (48/522)	9 (55/609)	8 (37/469)
Any adverse events, %	99	99	99
Rash	25	36	37
Anemia	49	38	39
Discontinuation due to adverse events	14	8^†^	18

^†^During telaprevir/placebo phase.

NA: not available; RGT: response-guided therapy; ITT: intention to treat.

**Table 4:** Stopping rules for PI-based therapy.

Timepoint	Criteria for stopping	Action
Telaprevir (TVR) [5]		

Week 4 or 12	HCV-RNA > 1000 IU/mL	Discontinue TVR/PEG-IFN/RBV
Week 24	HCV-RNA detectable	Discontinue PEG-IFN/RBV

Boceprevir (BOC) [6]		

Week 12	HCV-RNA ≥ 100 IU/mL	Discontinue BOC/PEG-IFN/RBV
Week 24	Confirmed, detectable HCV-RNA	Discontinue BOC/PEG-IFN/RBV

**Table 5:** Responses in treatment-experienced patients.

	Boceprevir (BOC)		Telaprevir (T)		
	Experienced		Experienced		
	RESPOND-2 [7] LI + BOC RGT + BOC/PR48 (ITT)		REALIZE [8] T12/PR48 + LI + T12/PR48 (ITT)		

Overall rates, %	Partial responder	Relapser	Null responder	Partial responder	Relapser

SVR	46	72	31	57	86
Relapse	13		26	23	7
Any adverse events, %	100		98		
Rash	15		37		
Anemia	44		33		
Discontinuation due to adverse events	10		13		

LI: lead-in; RGT: response-guided therapy; ITT: intention to treat.

**Table 6:** RGT Guidelines: Treatment-Naïve Patients.

When to Evaluate HCV-RNA Results		
	Boceprevir [6]	

At Treatment Week 8 and Week 12	At Treatment Week 24	Recommendation

Undetectable	Undetectable	Complete three-medicine regimen at TW28
Detectable	Undetectable	(1) Continue all three medicines and finish through TW36; and then (2) Administer peginterferon alfa and ribavirin and finish through TW48

	Telaprevir [5]	

At Treatment Week 4 and Week 12	Recommendation	

Undetectable	Receive an additional 12 weeks of PEG-IFN/RBV alone for total treatment duration of 24 weeks	
Detectable	Receive an additional 36 weeks of PEG-IFN/RBV alone for a total treatment duration of 48 weeks	

TW: treatment week.

**Table 7:** Boceprevir Dosing in Treatment-Experienced Patients [6].

Boceprevir		HCV Genotype 1 Dosing	
Previous Partial Responders or Relapsers Without Cirrhosis		PEG-IFN/RBV for 4 weeks, then BOC 800 mg (four 200 mg capsules) orally 3 times daily (every 7–9 hours) with food [meal/light snack] to PEG-IFN/RBV regimen after 4 weeks of treatment—then RGT	

Previous Partial Responders or Relapsers With Compensated Cirrhosis			
OR		4 weeks PEG-IFN/RBV followed by 44 weeks BOC 800 mg 3 times daily in combination with PEG-IFN/RBV	
<2-log_10_ HCV-RNA decline by treatment week 12 during prior therapy with PEG-IFN/RBV			

Response-Guided Therapy	Assessment (HCV-RNA Results)		Recommendation
	At Treatment Week 8	At Treatment Week 24

Previous partial responders or relapsers	Undetectable	Undetectable	Complete 3-medicine regimen at TW36
	Detectable	Undetectable	(1) Continue all 3 medicines and finish through TW36 and then:
			(2) Administer PEG-IFN/RBV and finish through TW48

BOC: boceprevir; TW: treatment week.

**Table 8:** Telaprevir Dosing in Treatment-Experienced Patients [5].

Telaprevir 750 mg taken orally 3 times a day (7–9 hours apart) with food in combination with PEG-IFN/RBV for 12 weeks			
Prior Relapse Patients	TVR/PEG-IFN/RBV	PEG-IFN/RBV	Total Duration

HCV RNA undetectable at weeks 4 and 12	First 12 weeks	Additional 12 weeks	24 weeks
HCV-RNA detectable (1000 IU/mL or less) at weeks 4 and/or 12	First 12 weeks	Additional 36 weeks	48 weeks
Prior Partial and Null Responder Patients	First 12 weeks	Additional 36 weeks	48 weeks

**Table 9:** PI Dosing Recommendations for G1 Patients [5, 6].

Medication	Dosing
Boceprevir (BOC)* Treatment-naïve without cirrhosis	PEG-IFN/RBV for 4 weeks, then BOC 800 mg (four 200 mg capsules) orally 3 times daily (every 7–9 hours) with food [meal/light snack] to PEG-IFN/RBV regimen after 4 weeks of treatment—then RGT

Boceprevir* Treatment-naïve patients with cirrhosis	4 weeks PEG-IFN/RBV followed by 44 weeks BOC 800 mg (four 200 mg capsules) 3 times daily (every 7–9 hours) in combination with PEG-IFN/RBV

Telaprevir (TVR)**	TVR 750 mg (two 375 mg tablets) taken orally 3 times a day (7–9 hours apart) with food in combination with PEG-IFN/RBV for 12 weeks

^∗^No dosage adjustment required for patients with any degree of renal impairment or for mild, moderate, or severe hepatic impairment.

^∗∗^Not recommended for patients with moderate or severe hepatic impairment (Child-Pugh B or C, score ≥ to 7) or patients with decompensated liver disease.

RGT: response-guided therapy.

## Abbreviations

AASLD: American Association for the Study of Liver Diseases
AEs: Adverse events
AUC: Area under the curve
CNI: Calcineurin inhibitor
CPT: Child-Pugh-Turcott
DAA: Direct-acting antiviral agent
DDIs: Drug-drug interactions
DRESS: Drug Rash with Eosinophilia and Systemic Symptoms syndrome
eRVR: Extended rapid virologic response
ESA: Erythropoiesis-stimulating agent
FDA: US Food and Drug Administration
HCV: Hepatitis C virus
Hgb: Hemoglobin
IFN: Interferon
NNRTI: Nonnucleoside reverse transcriptase inhibitors
PEG-IFN: Pegylated interferon
PI: Protease inhibitor
RAV: Resistance-associated variants
RBV: Ribavirin
RGT: Response-guided therapy
RVR: Rapid viral response
SJS: Stevens Johnson Syndrome
SOC: Standard of care
SVR: Sustained virologic response.
