# Supplementary figures and images for: Hepatitis C Virus: A Critical Appraisal of New Approaches to Therapy
Source: Hepat Res Treat. 2012 Oct 8;2012:138302. doi: 10.1155/2012/138302 (PMC3472509; doi:10.1155/2012/138302)

**Supplemental Figures 2-25: HCV Council Voting Results**

| **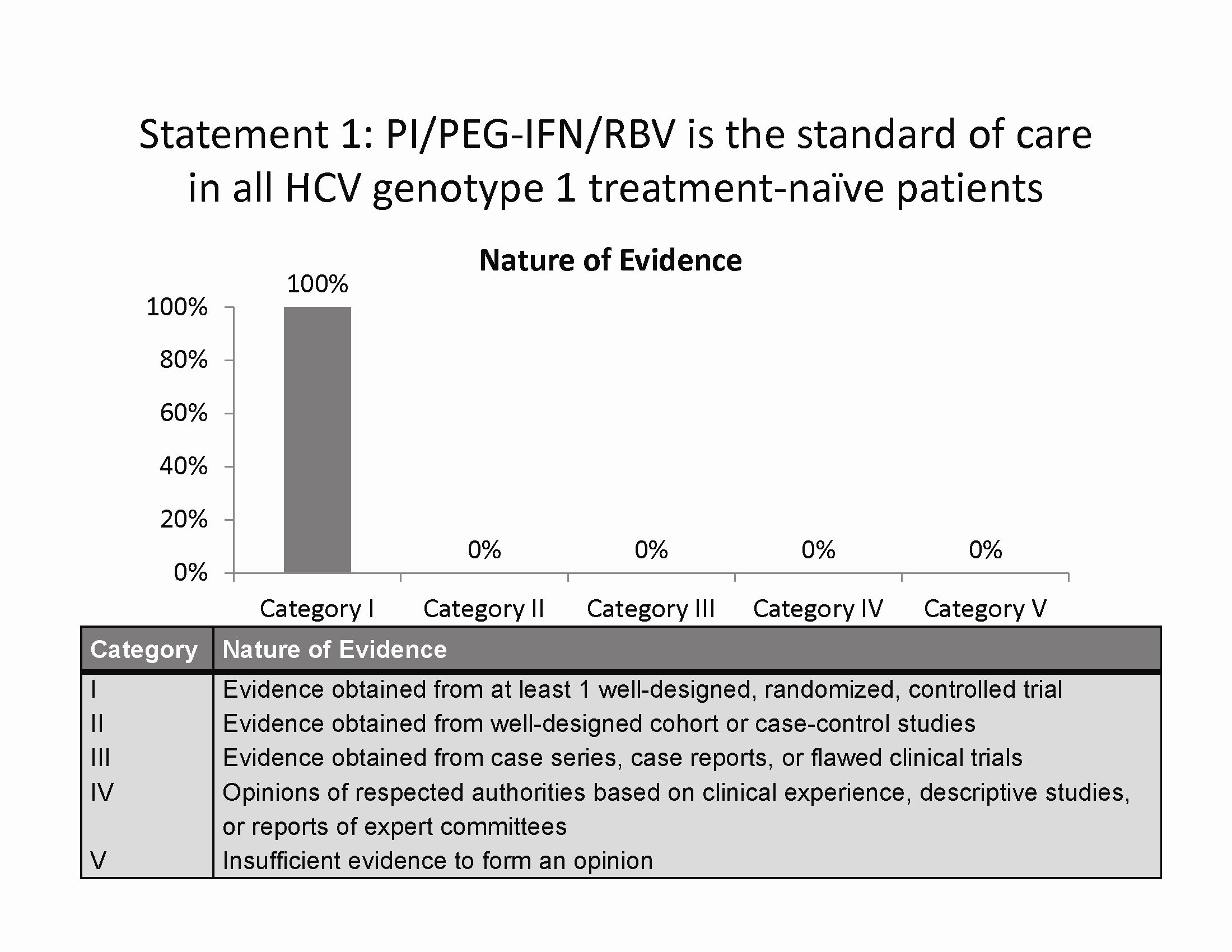** | **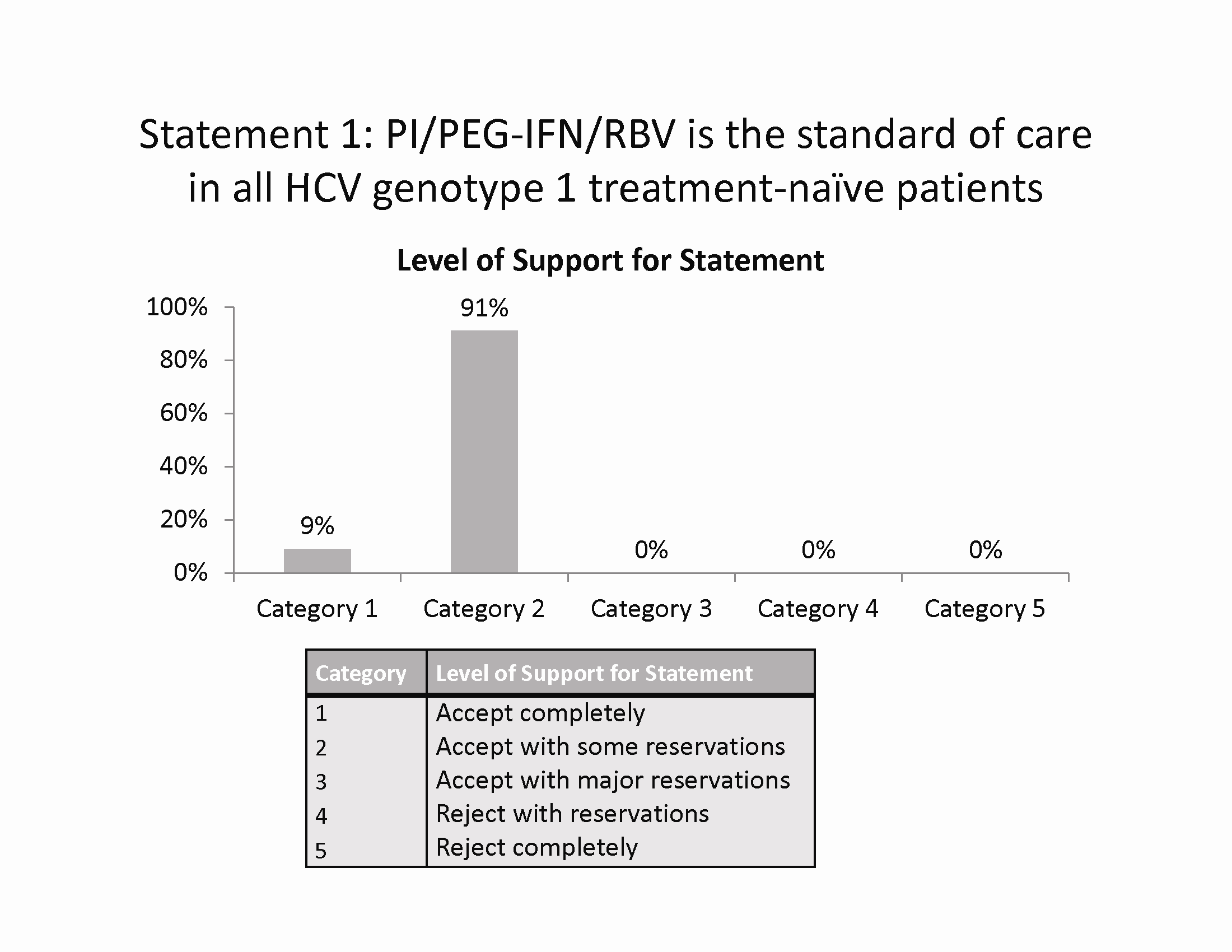** |
| --- | --- |
| **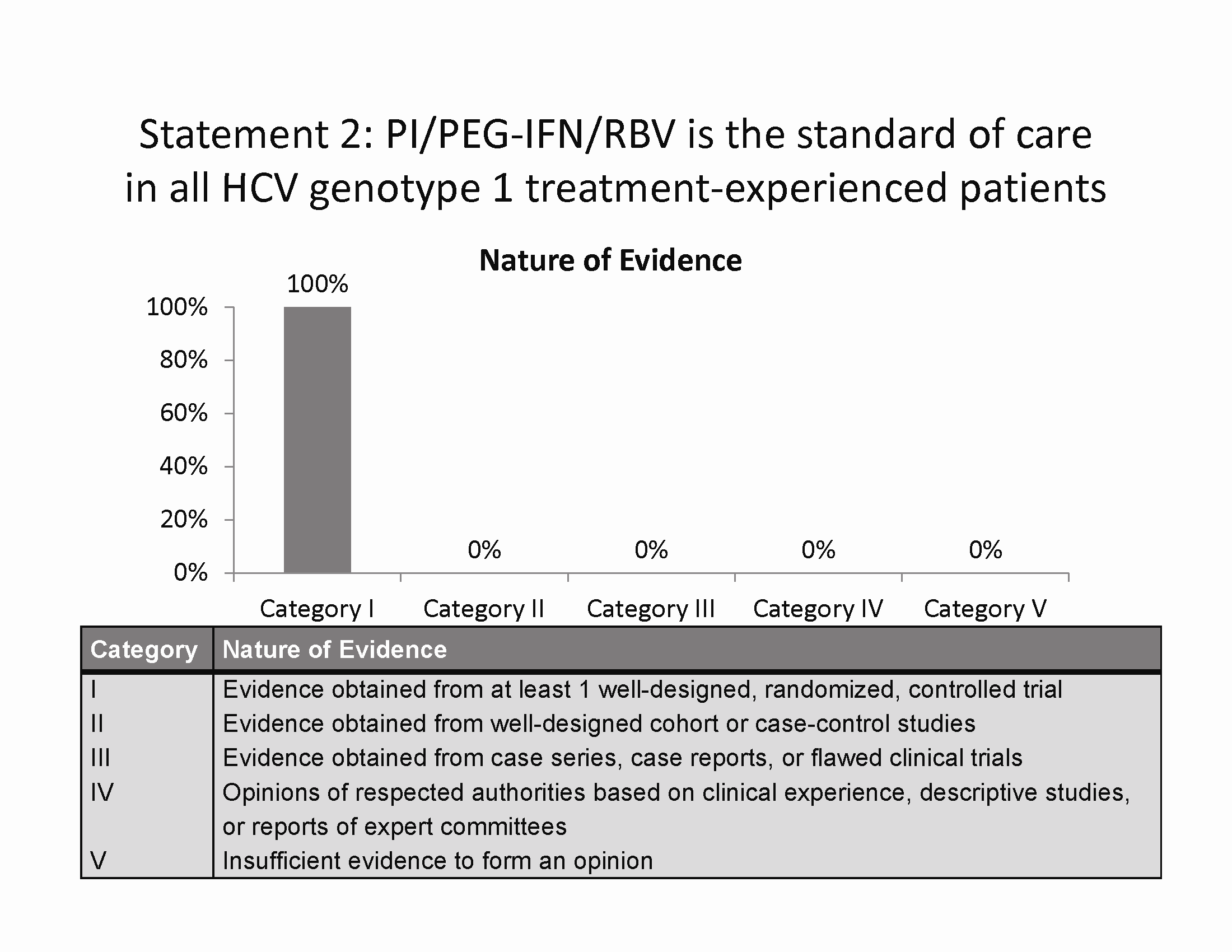** | **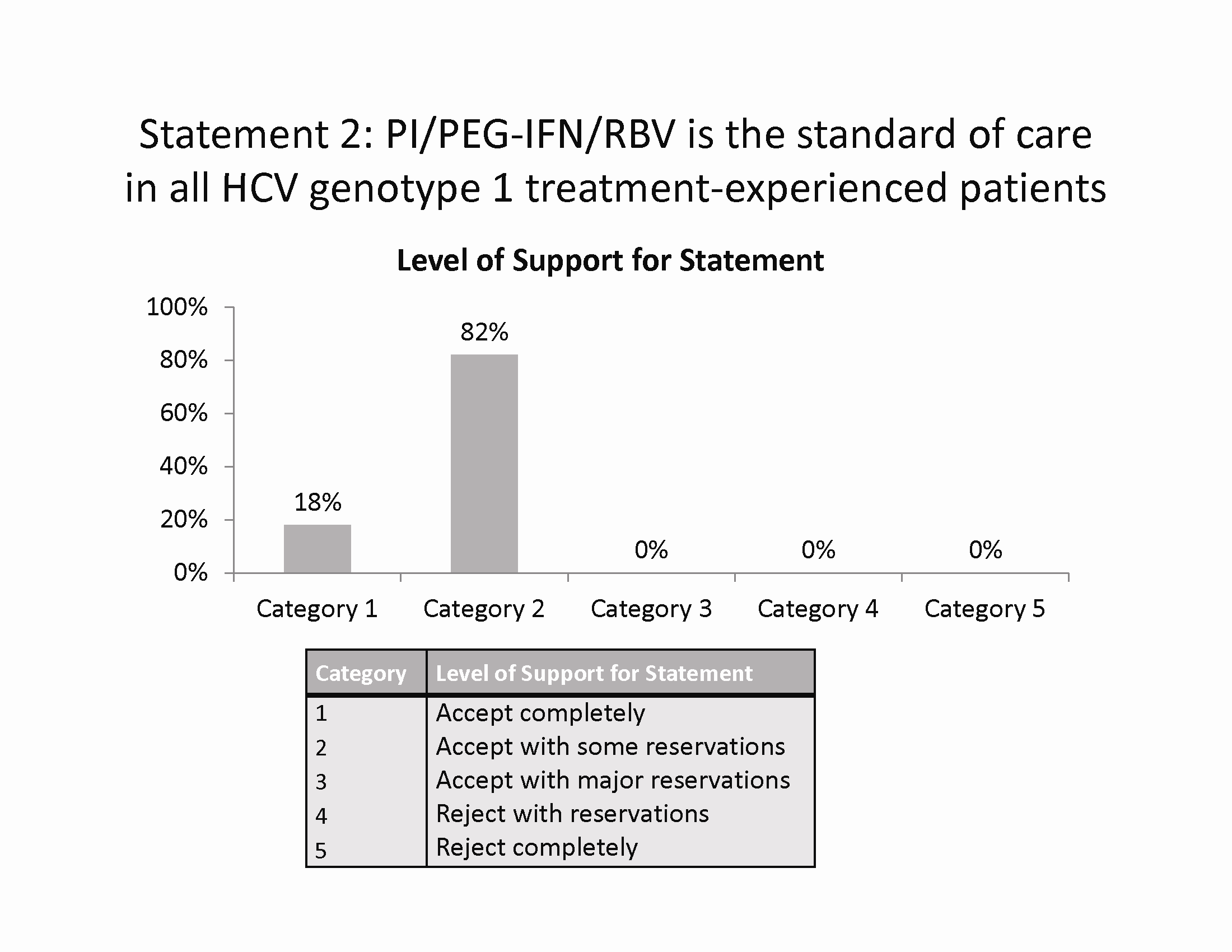** |
| **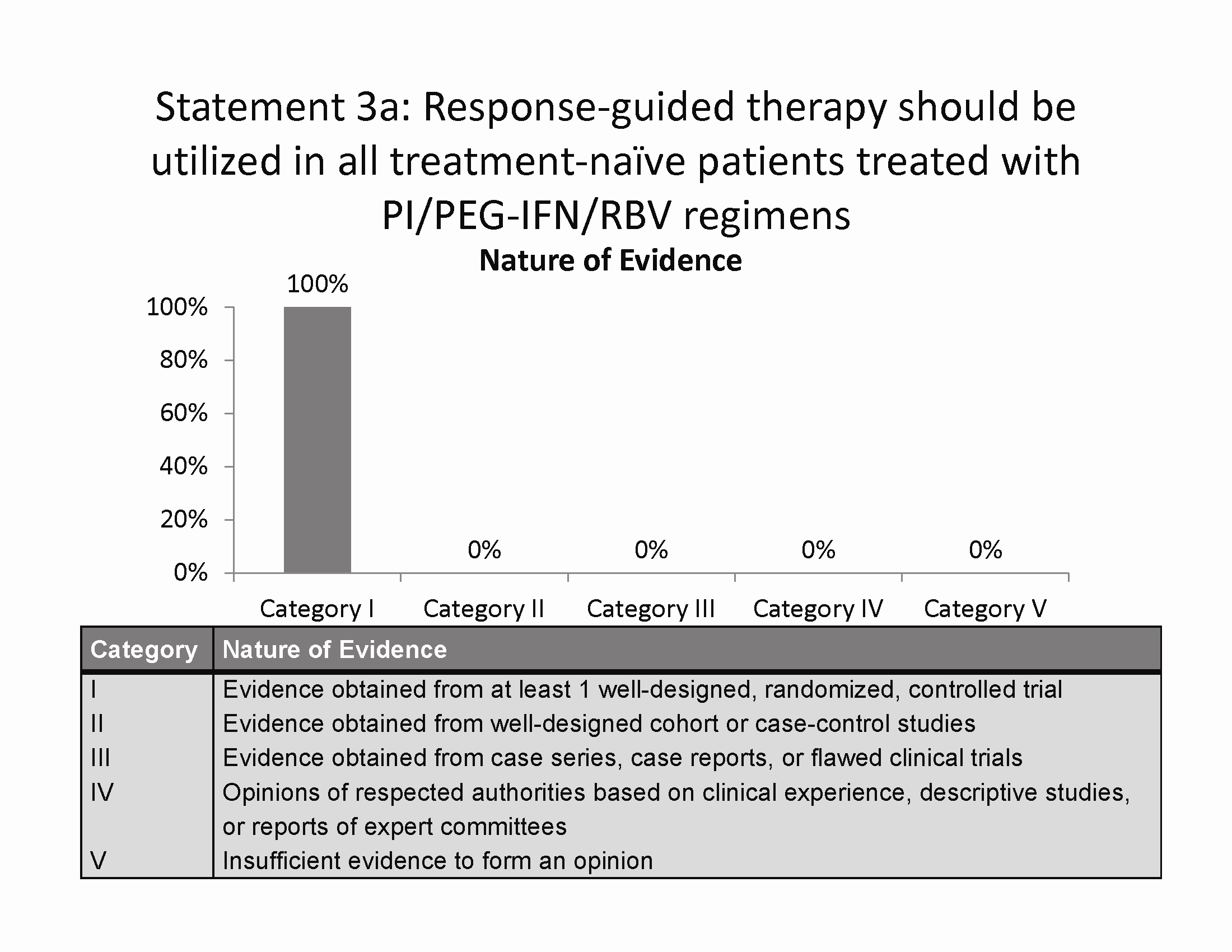** | **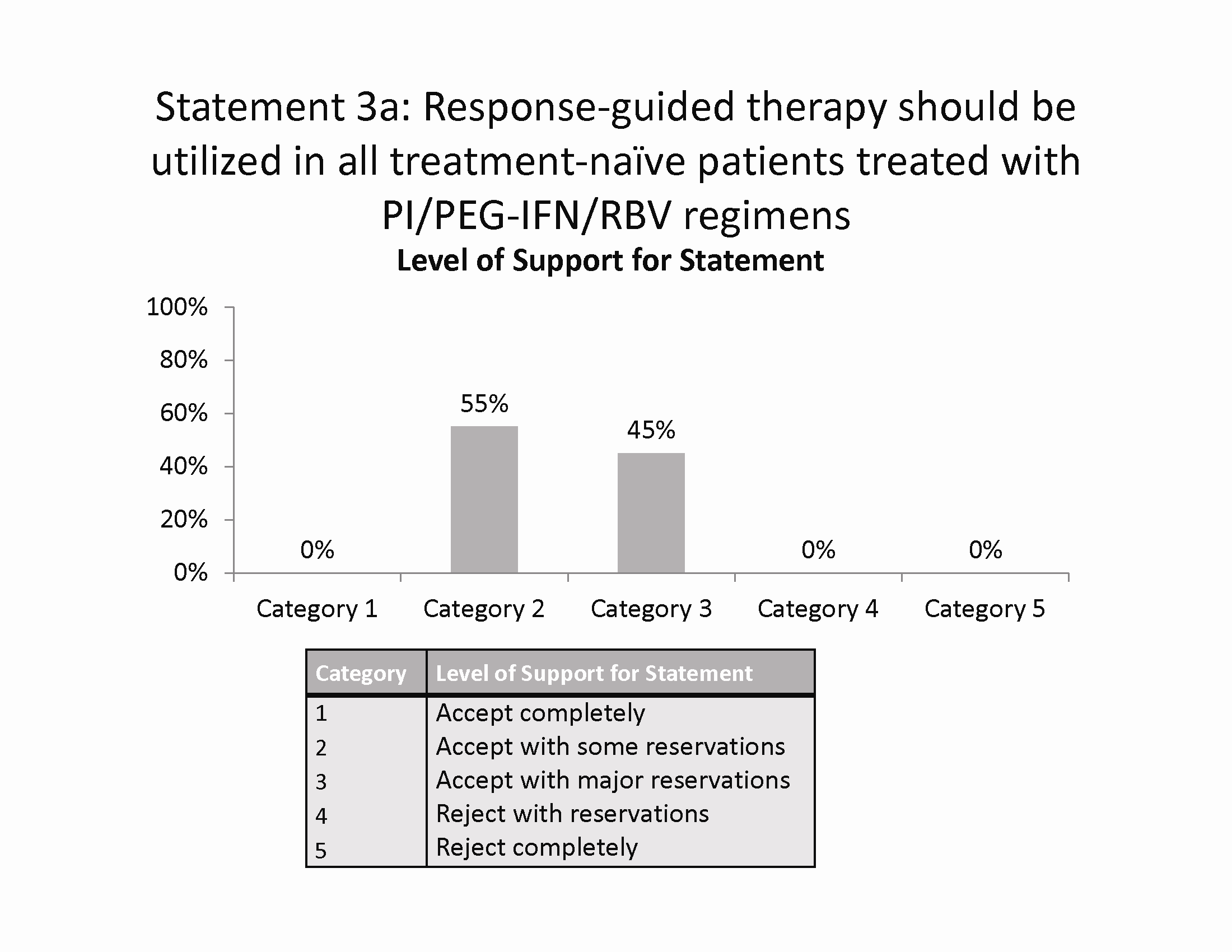** |
| **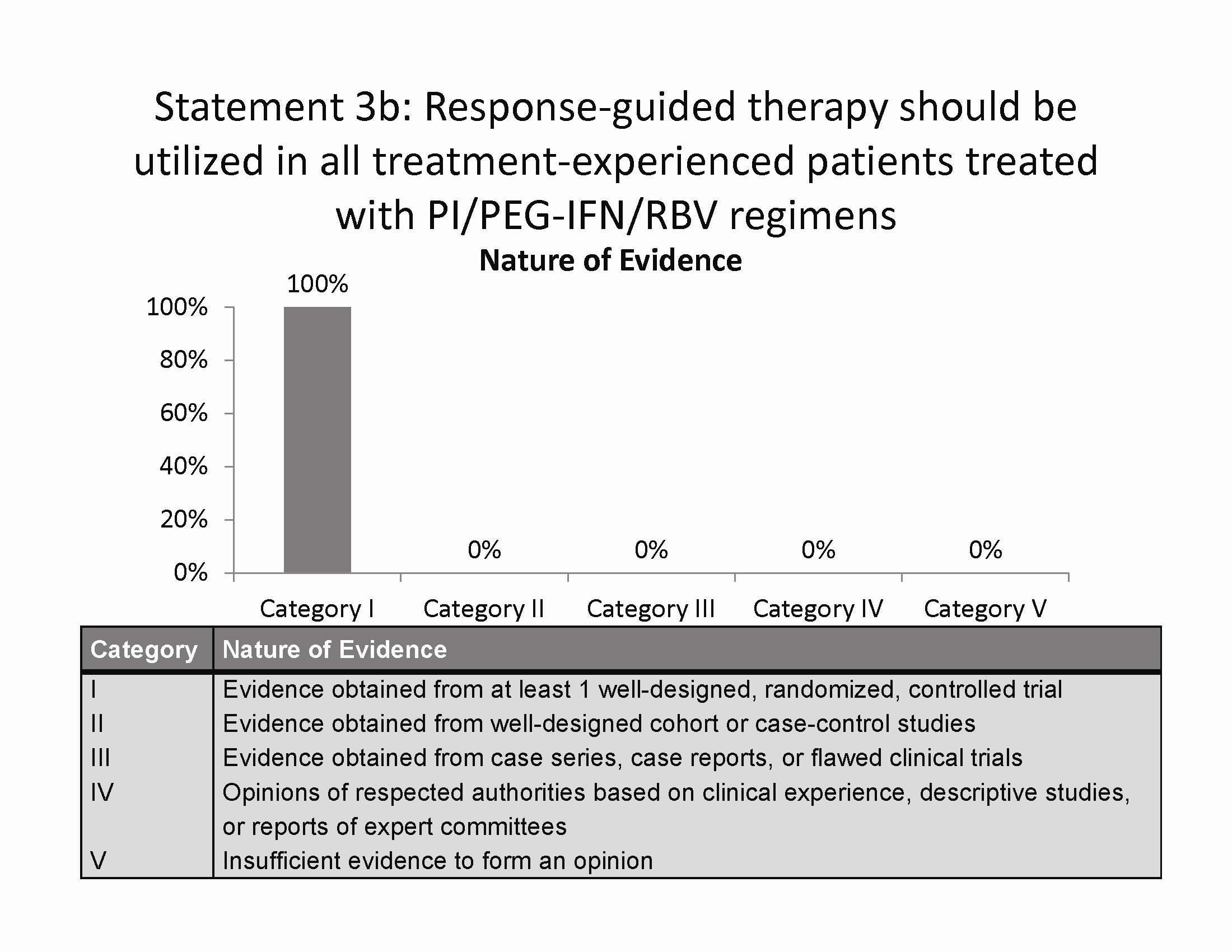** | **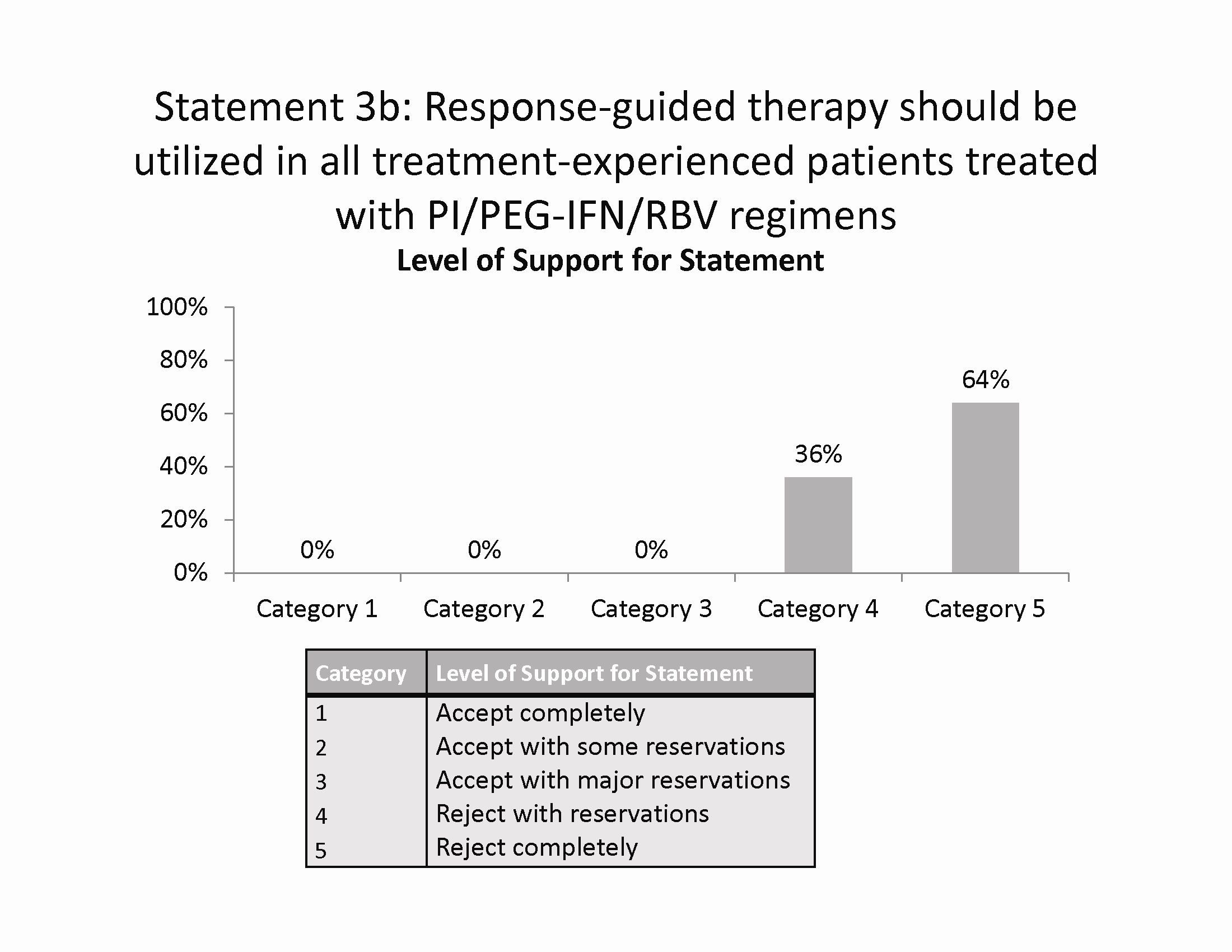** |
| **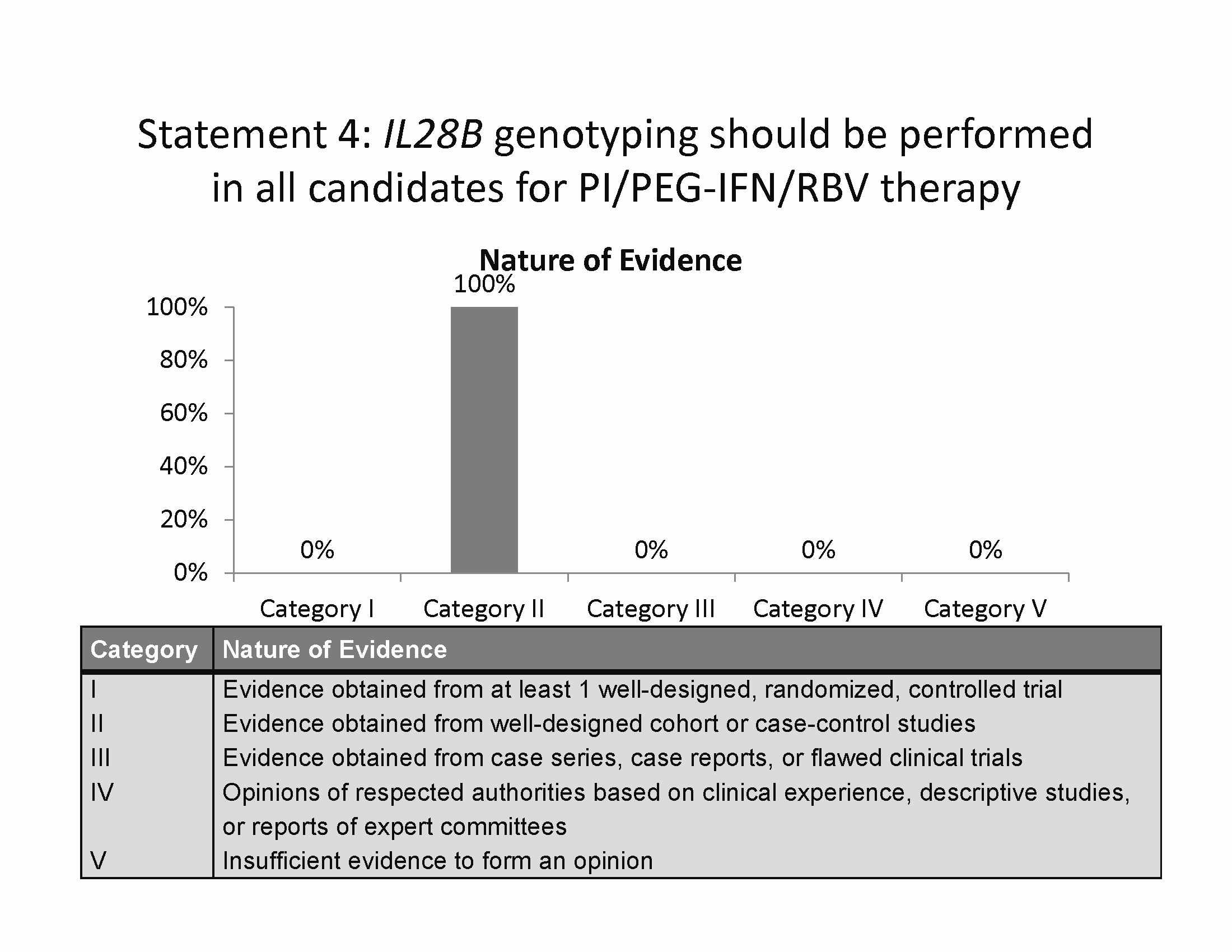** | **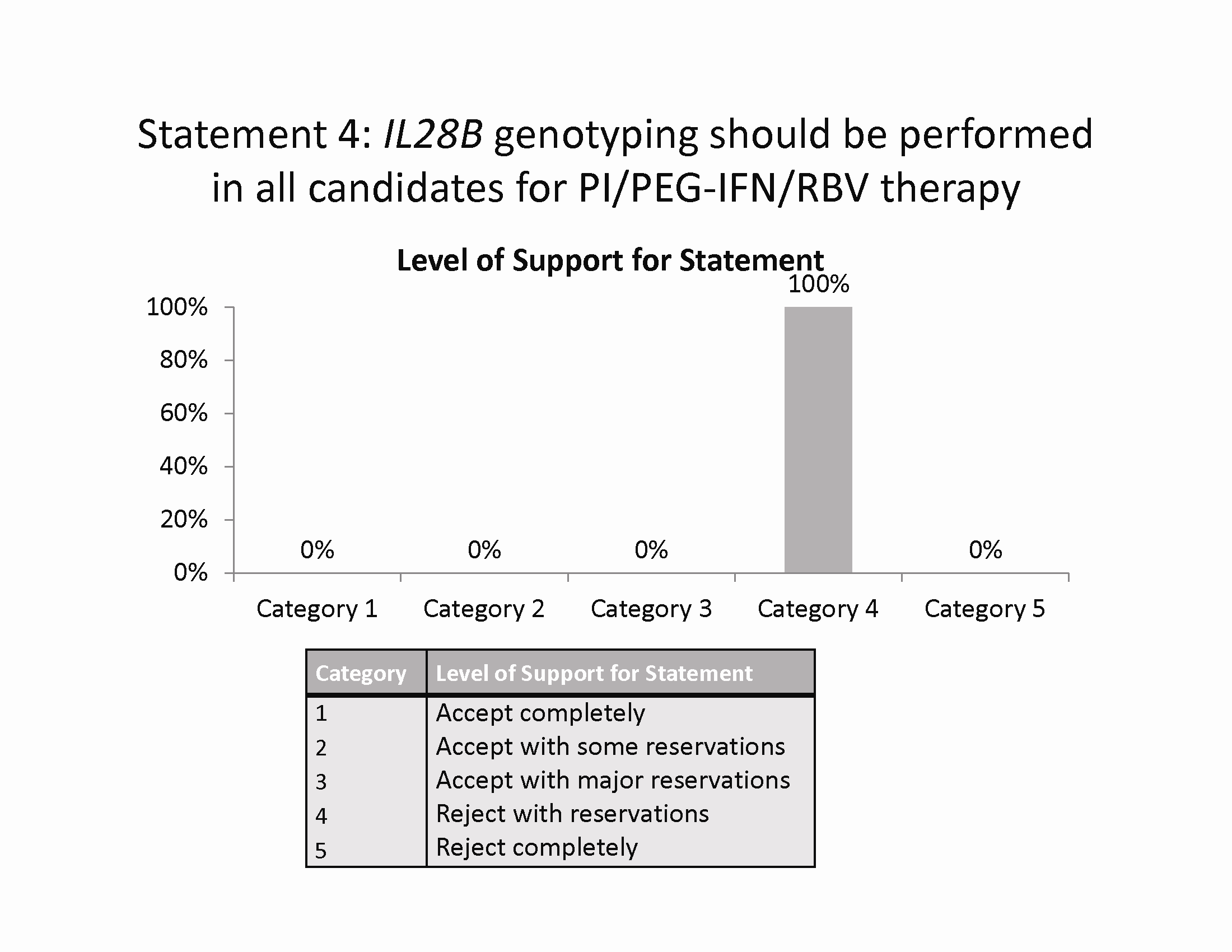** |
| **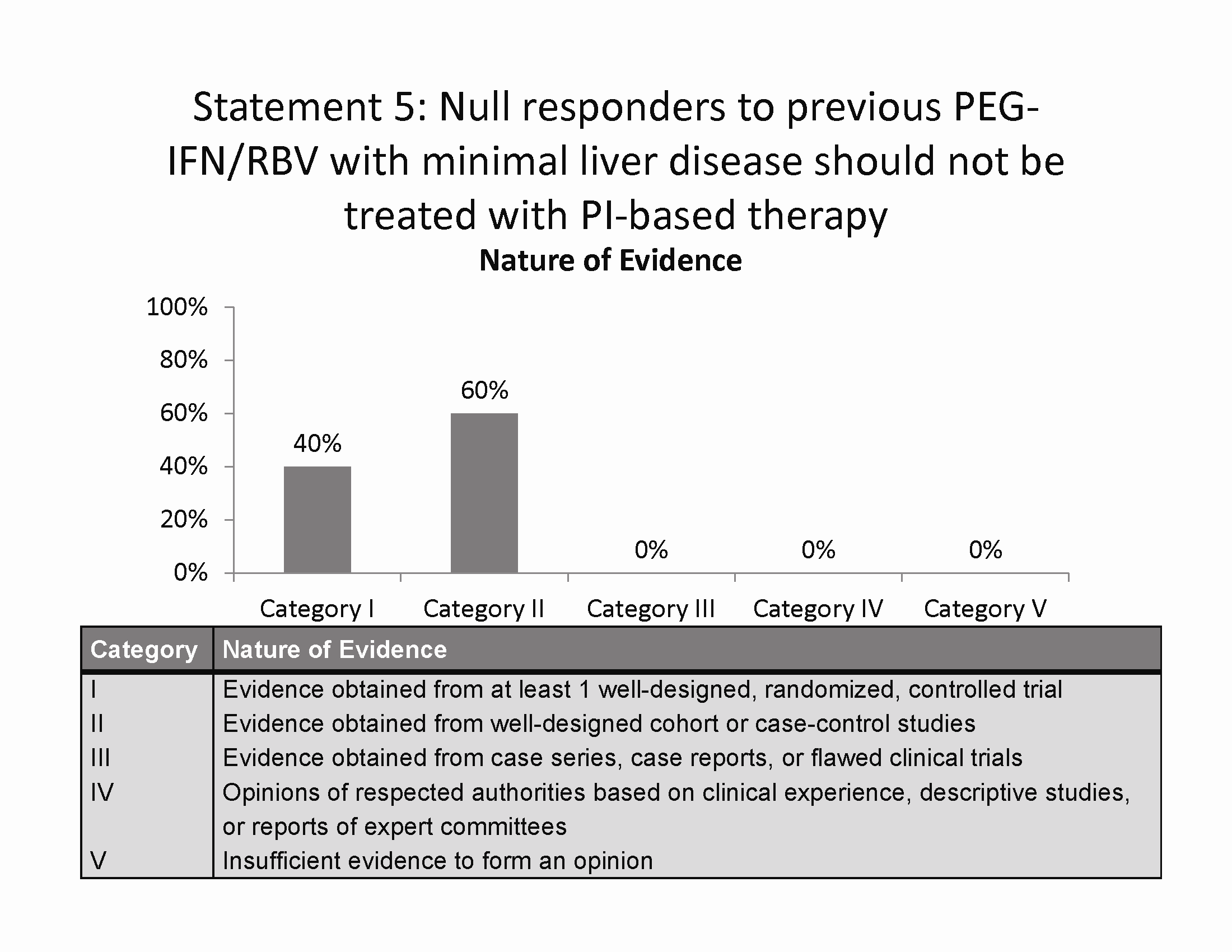** | **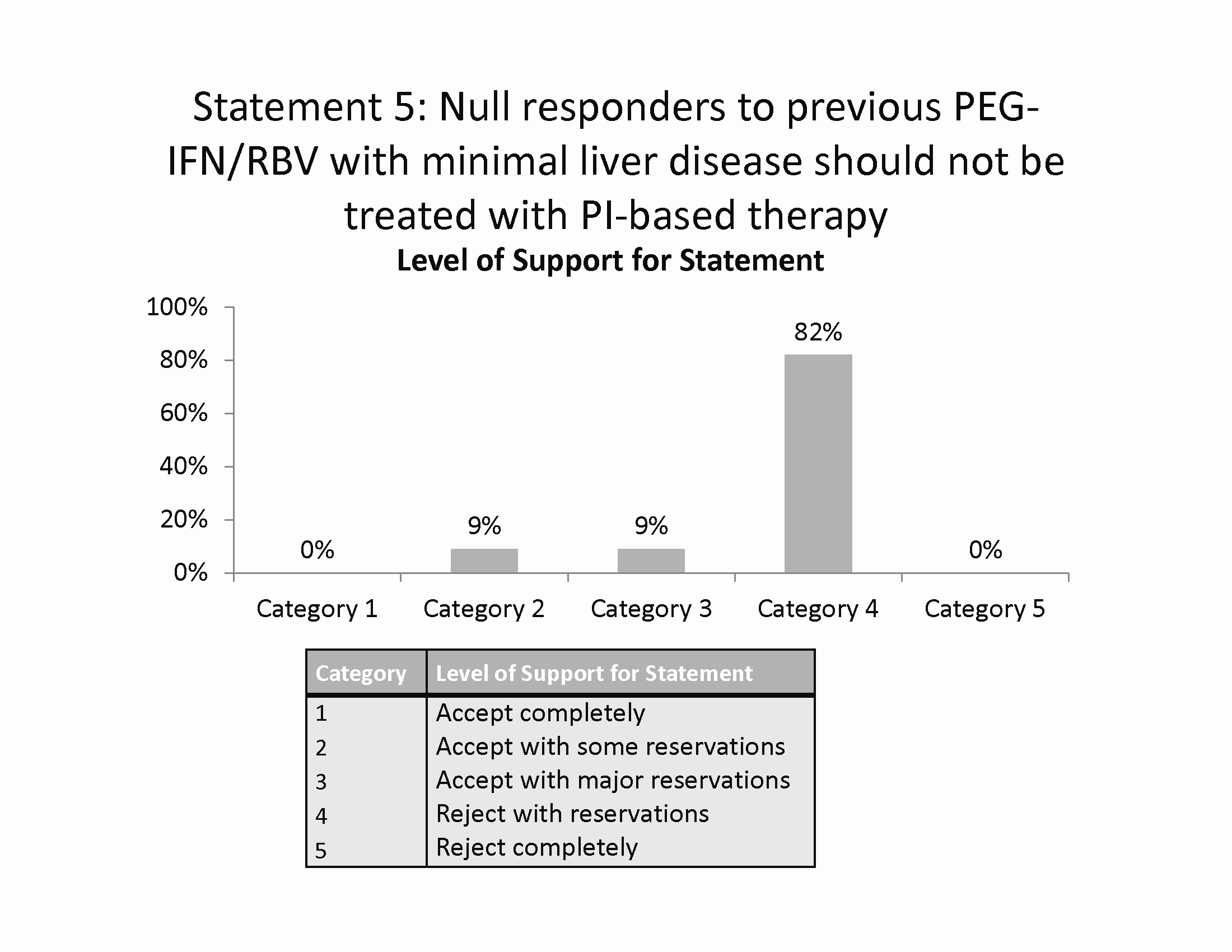** |
| **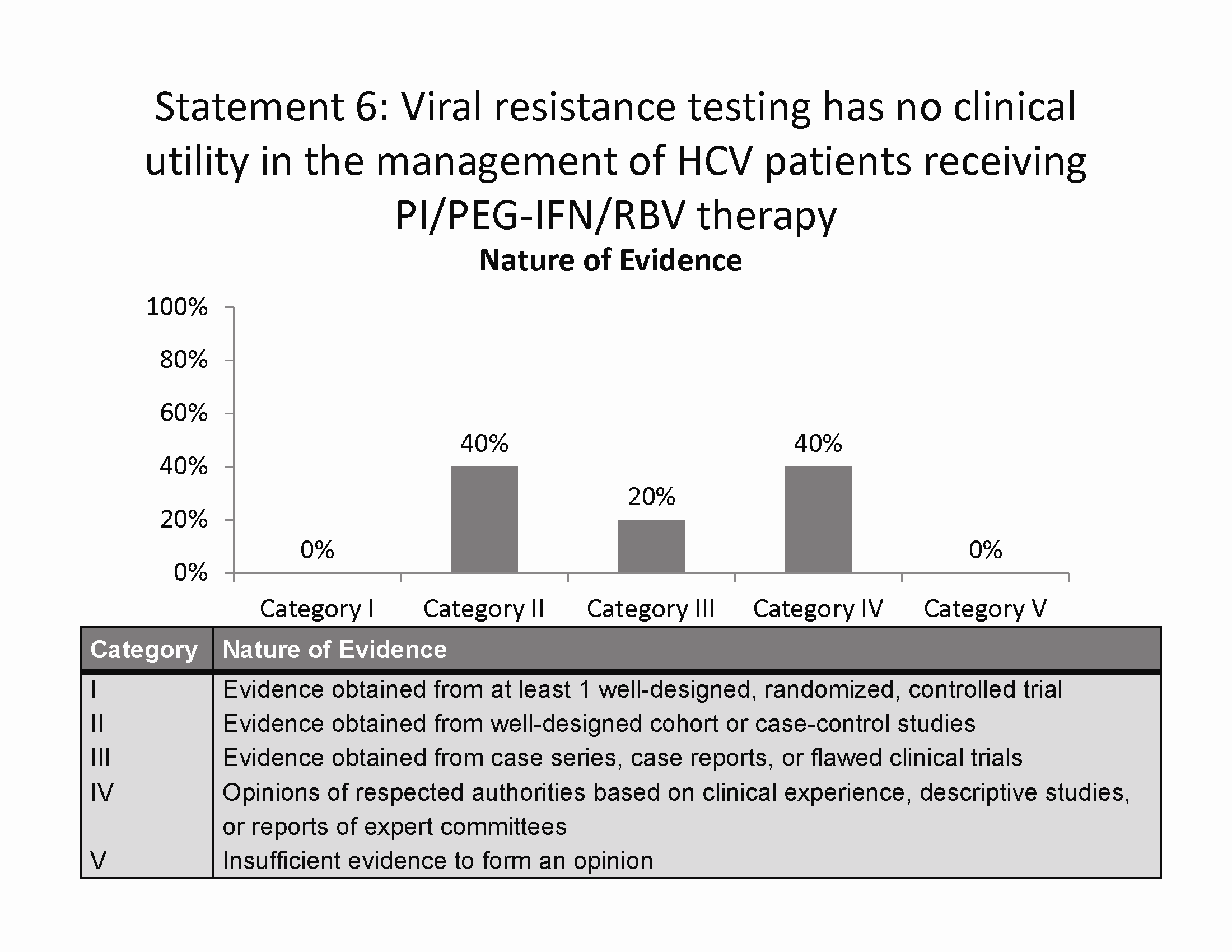** | **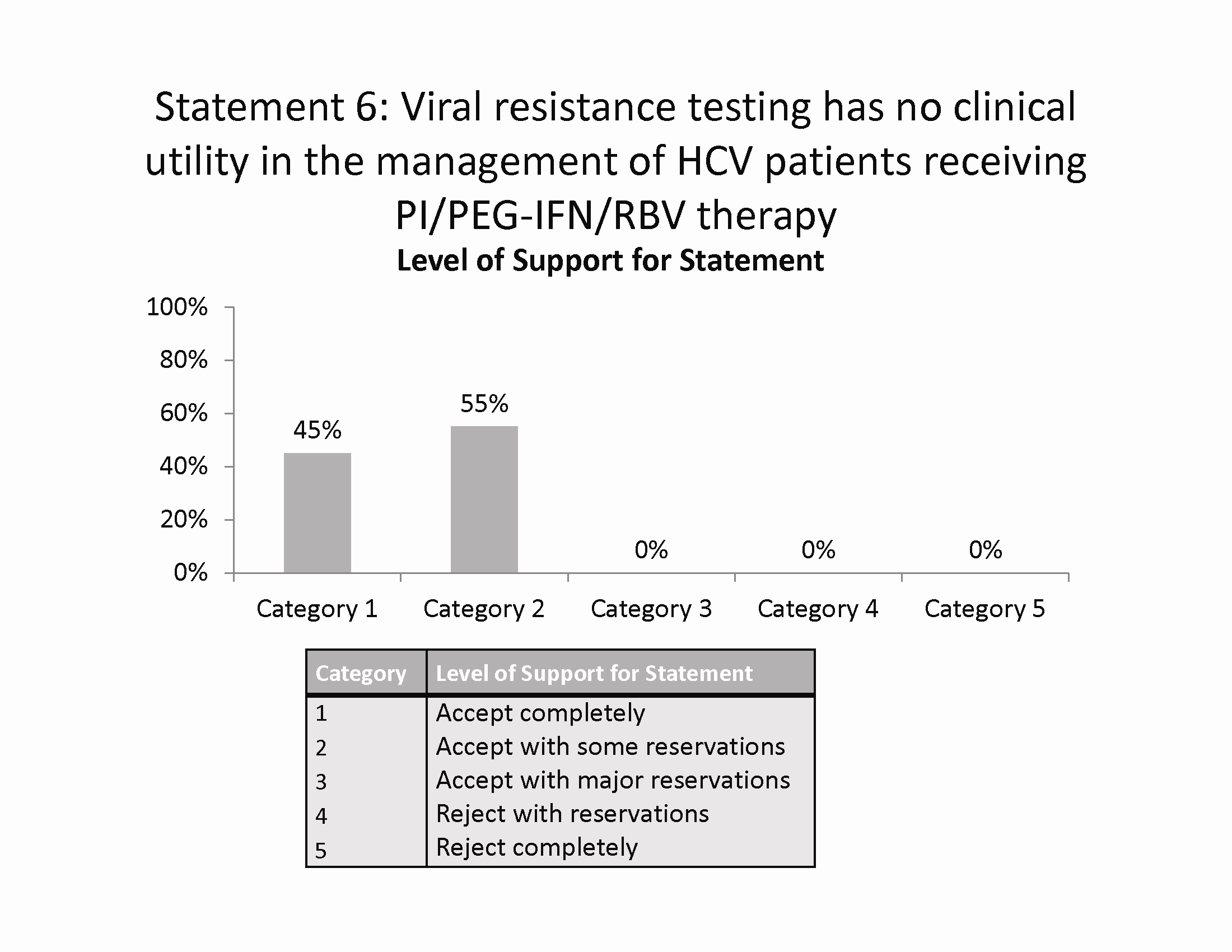** |
| **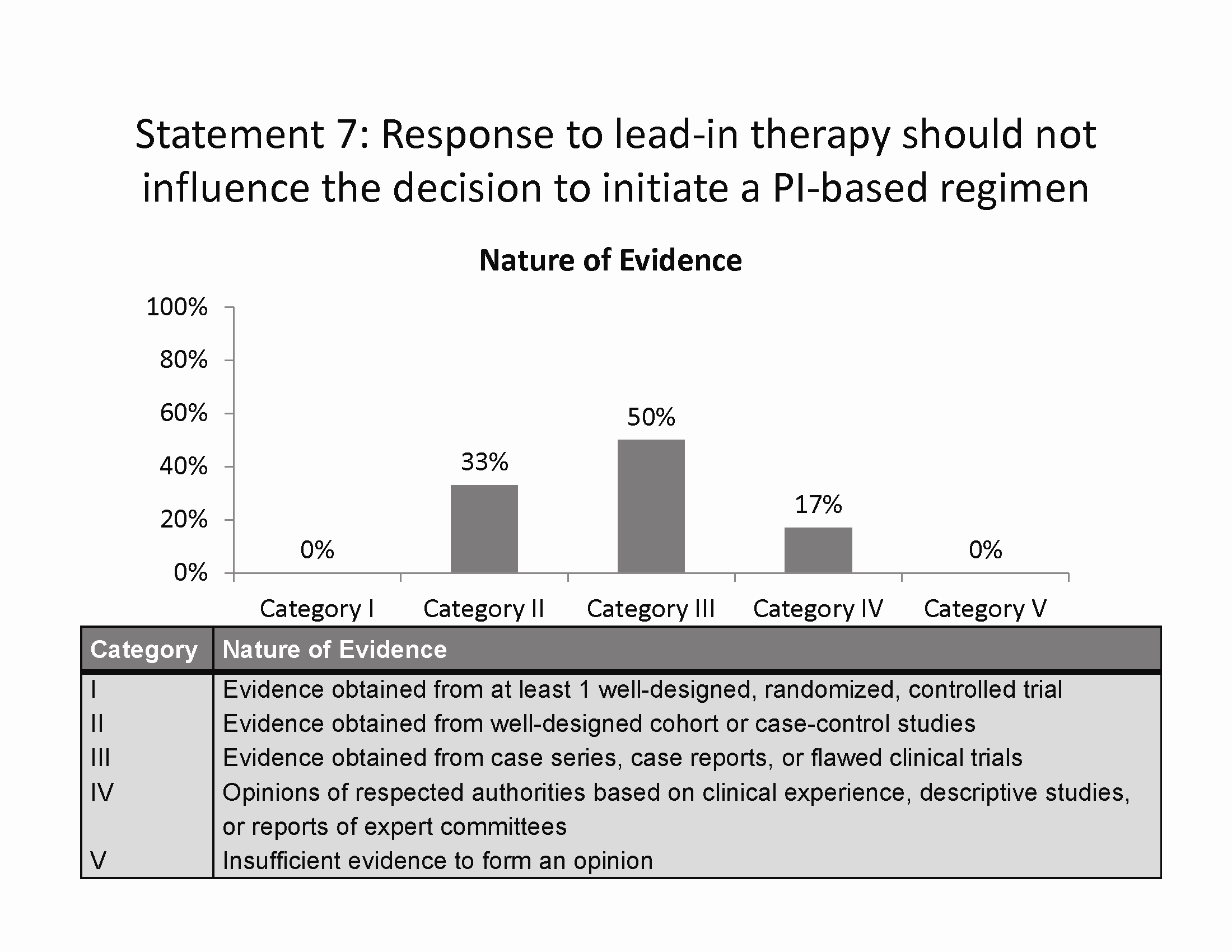** | **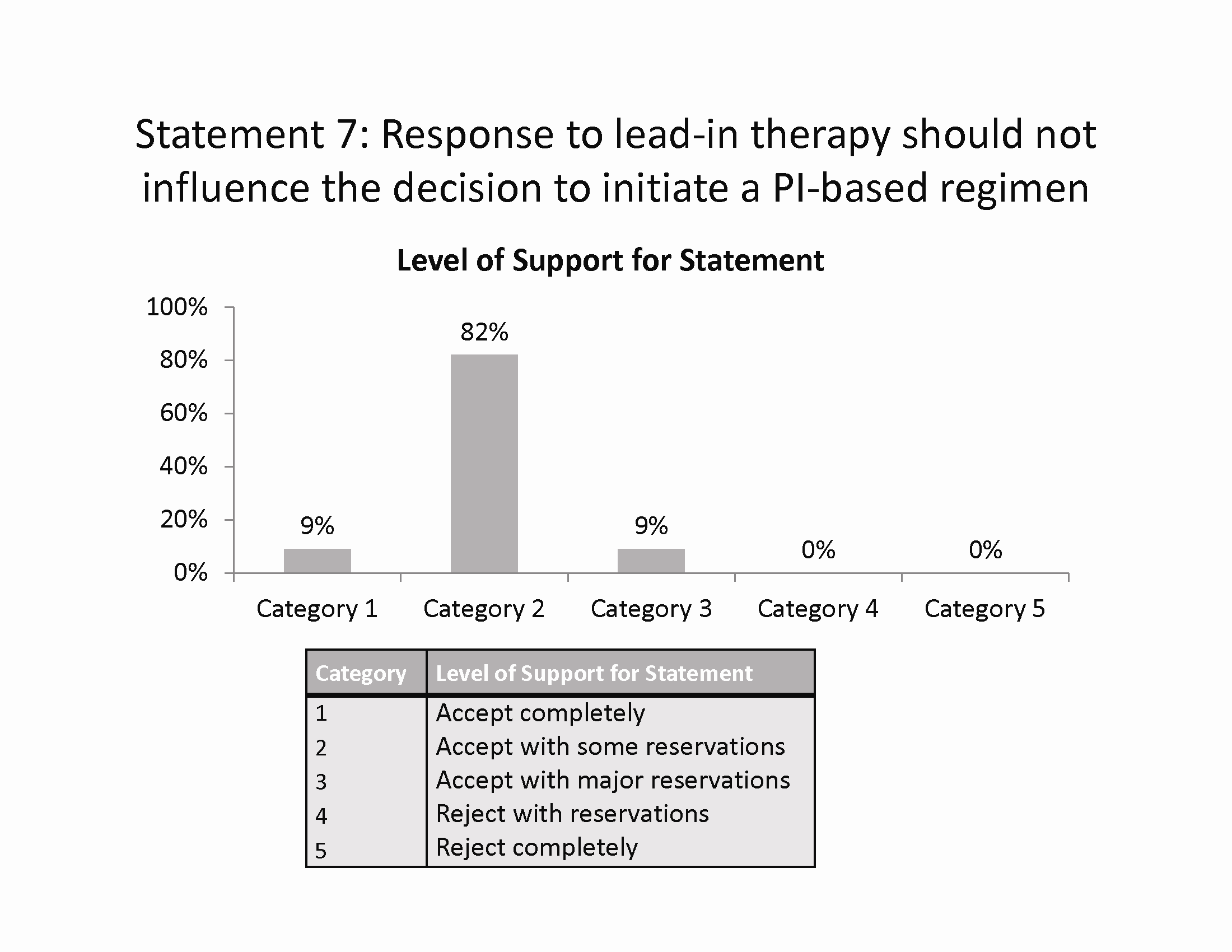** |
| **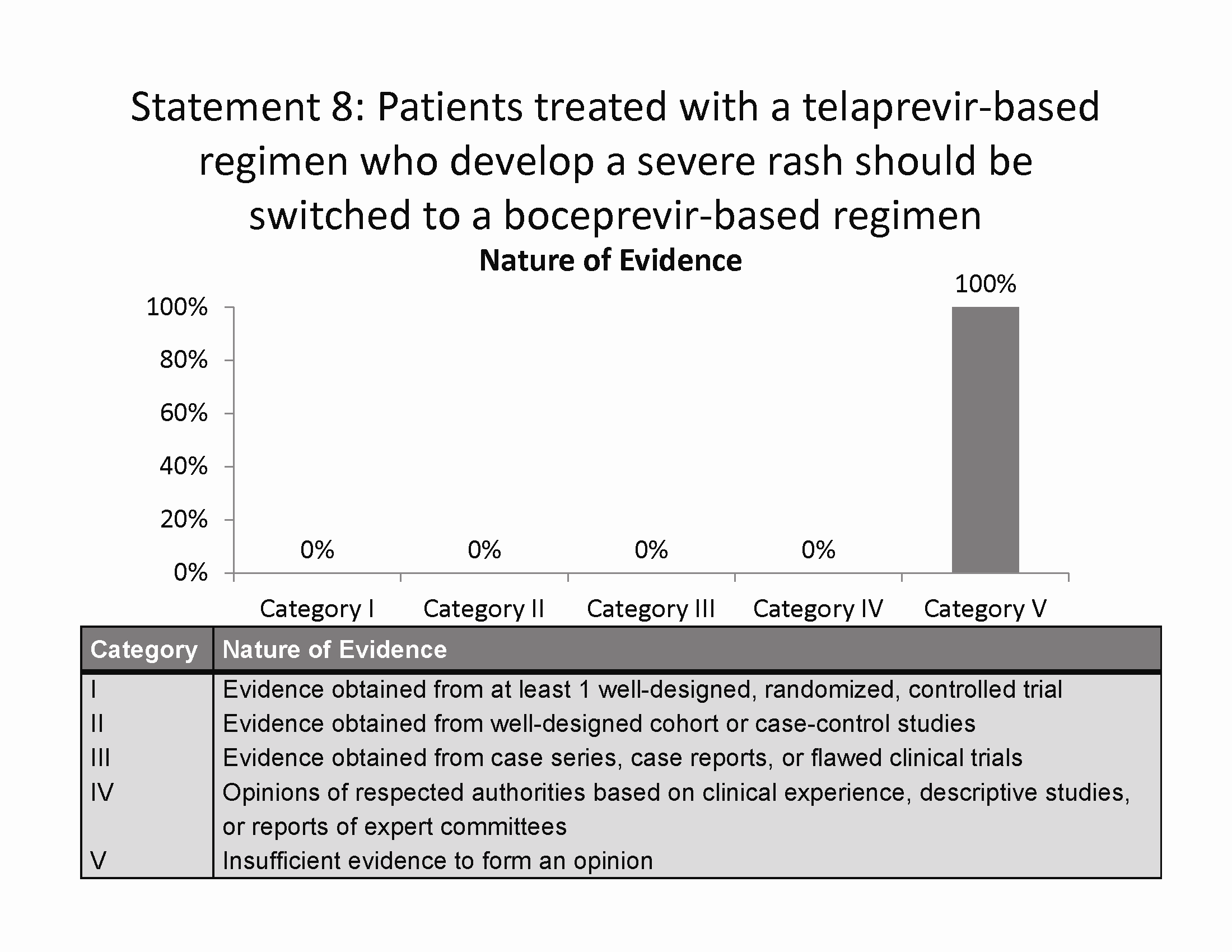** | **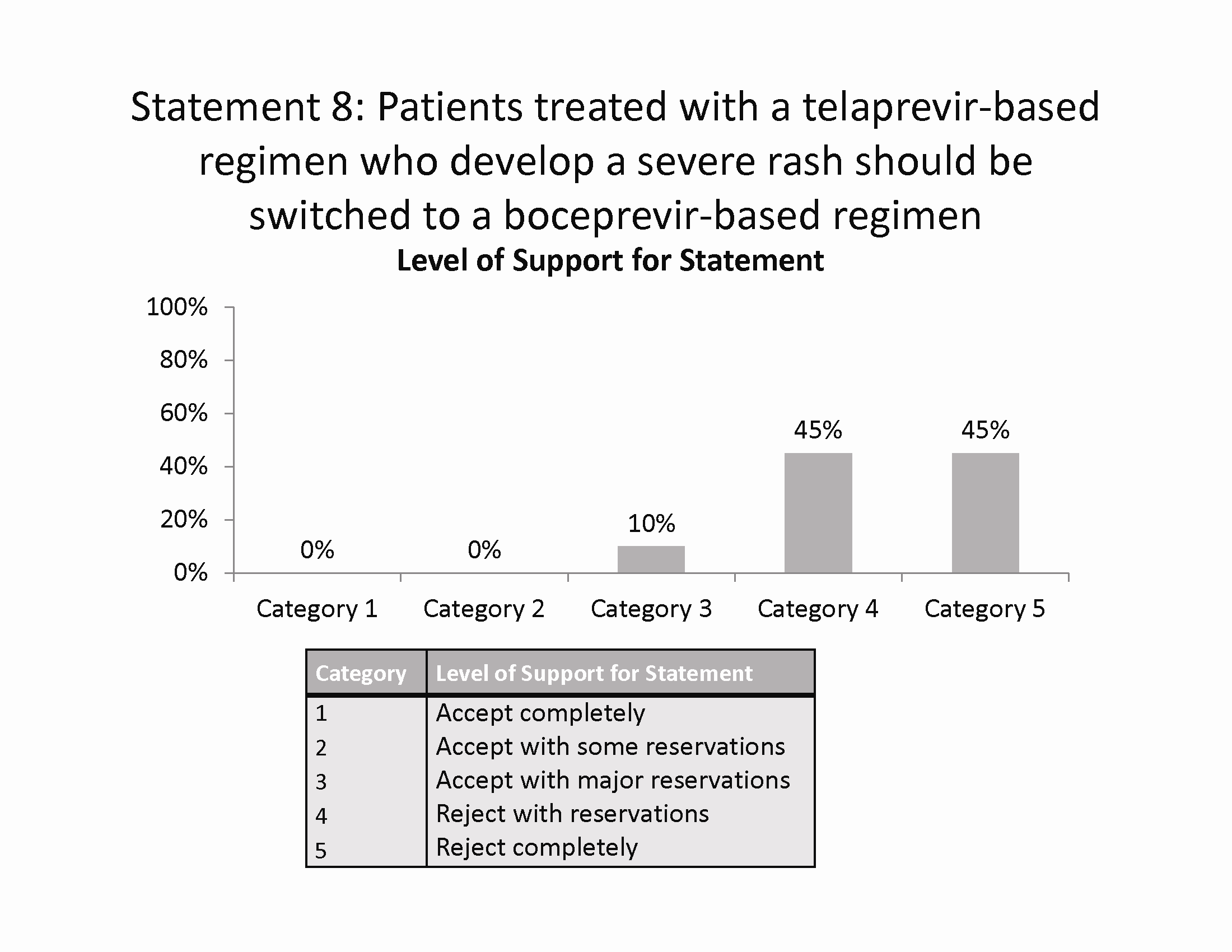** |
| **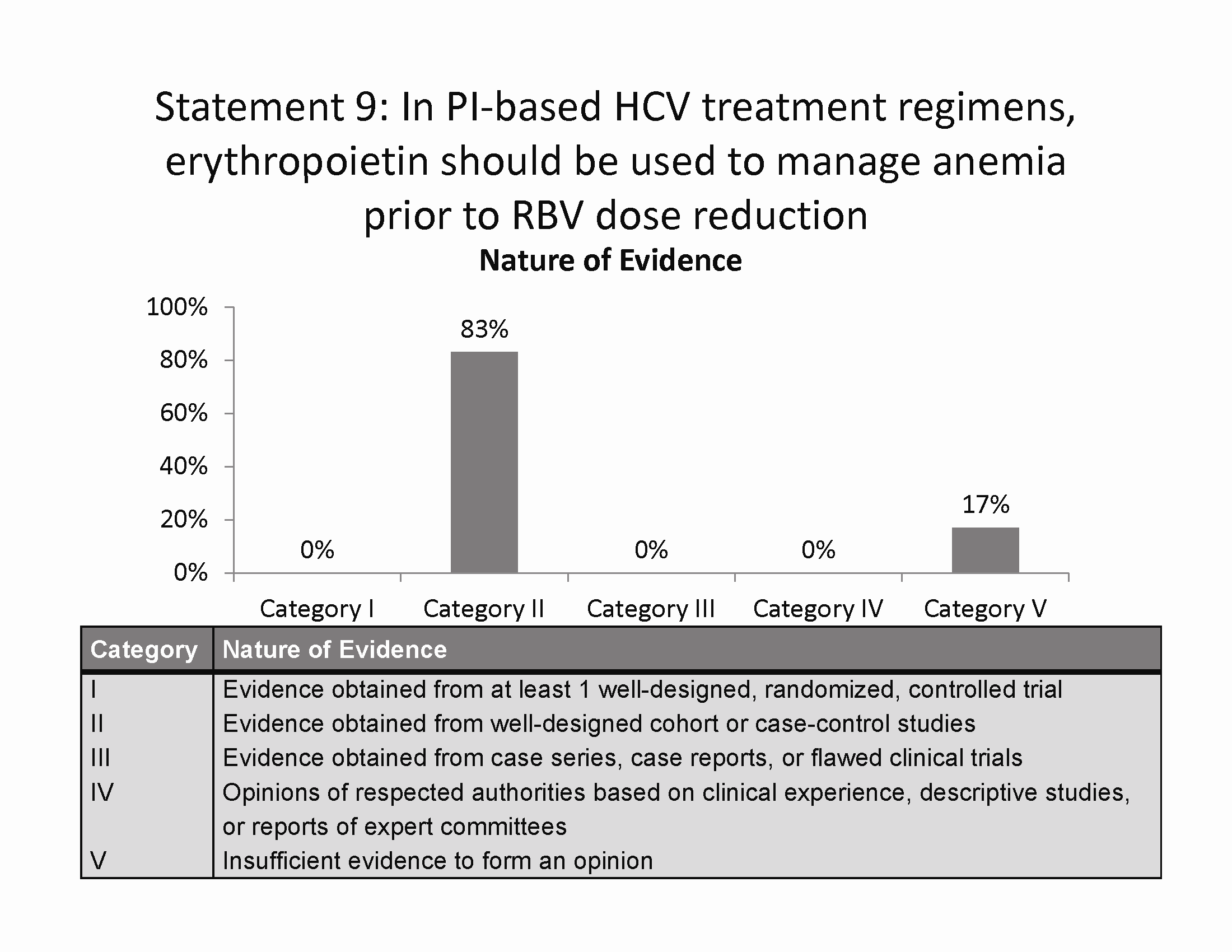** | **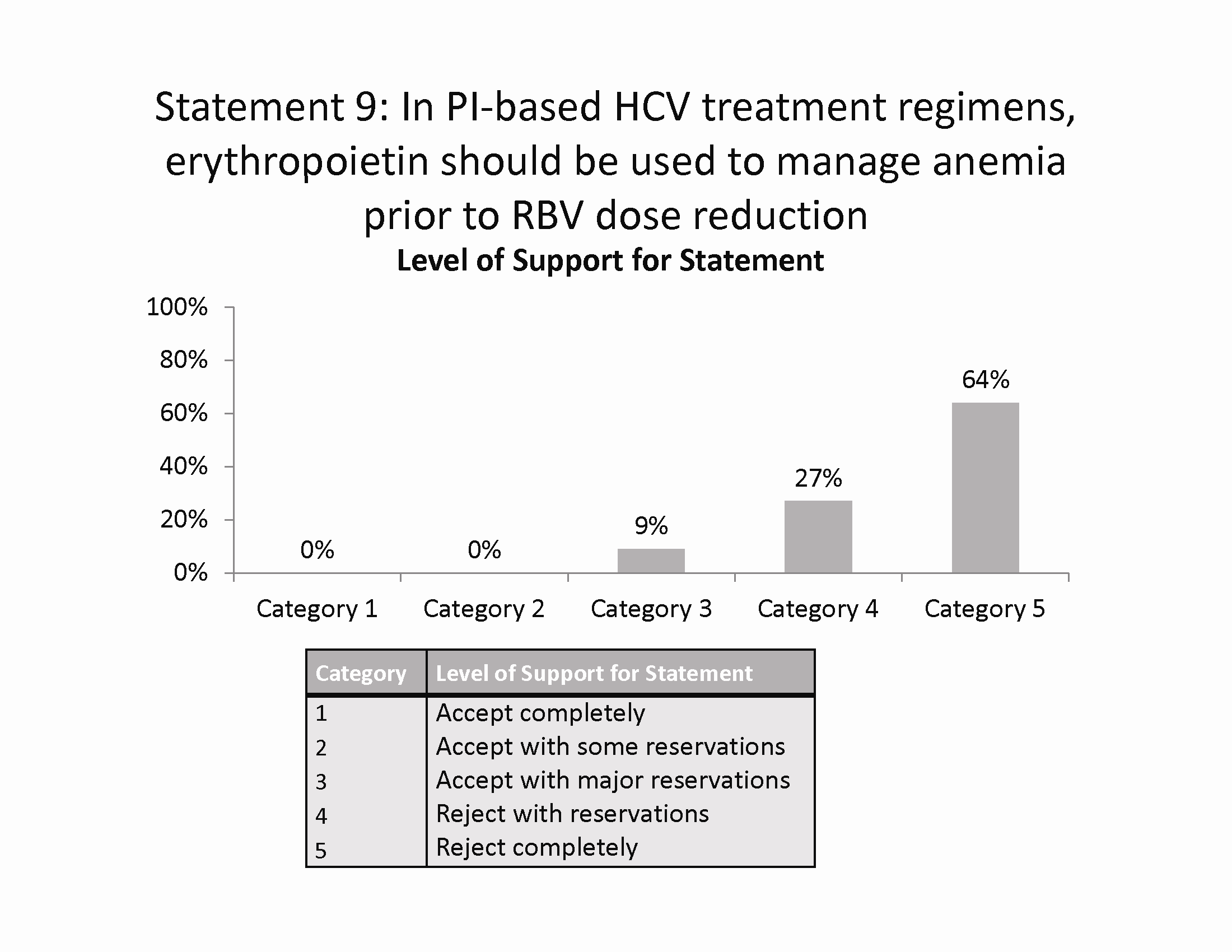** |
| **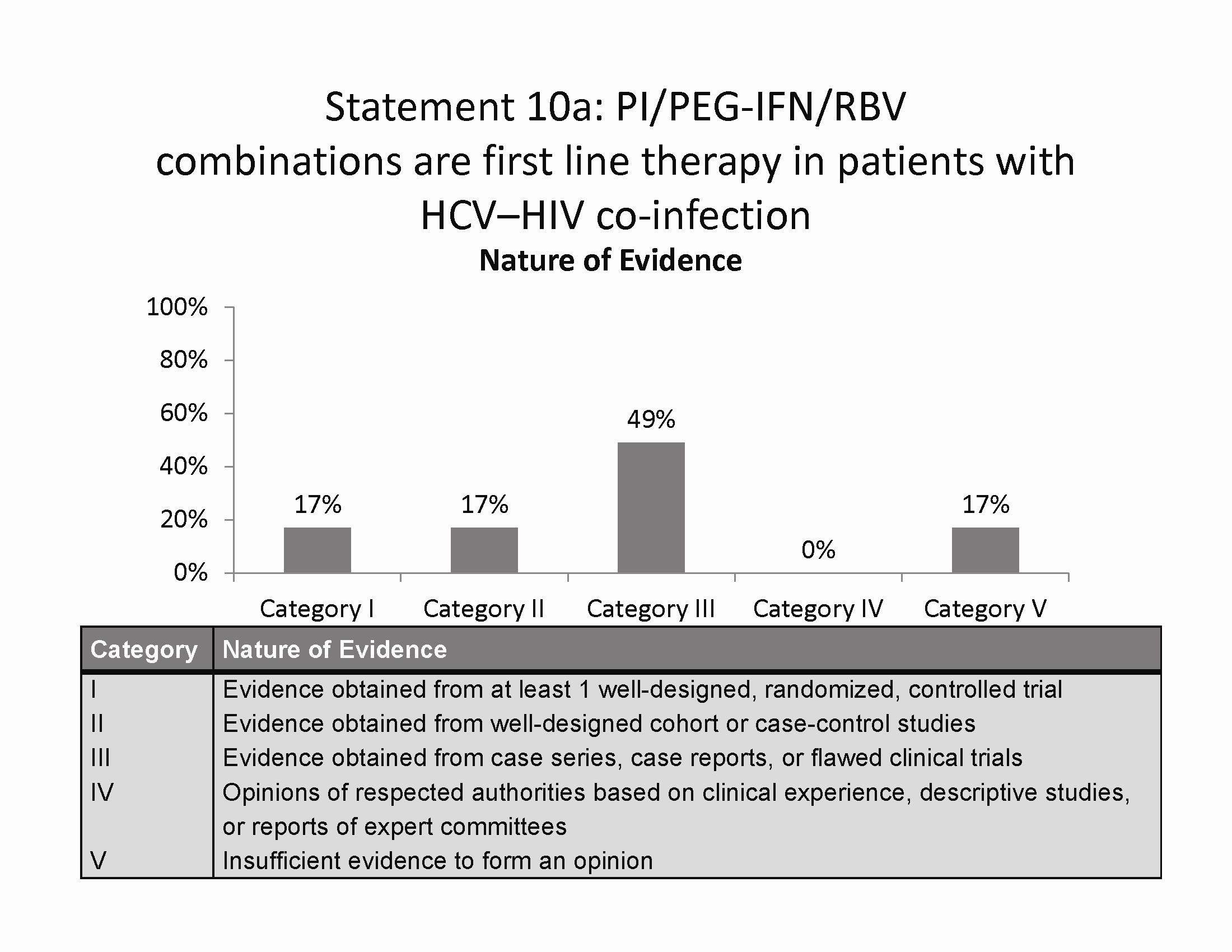** | **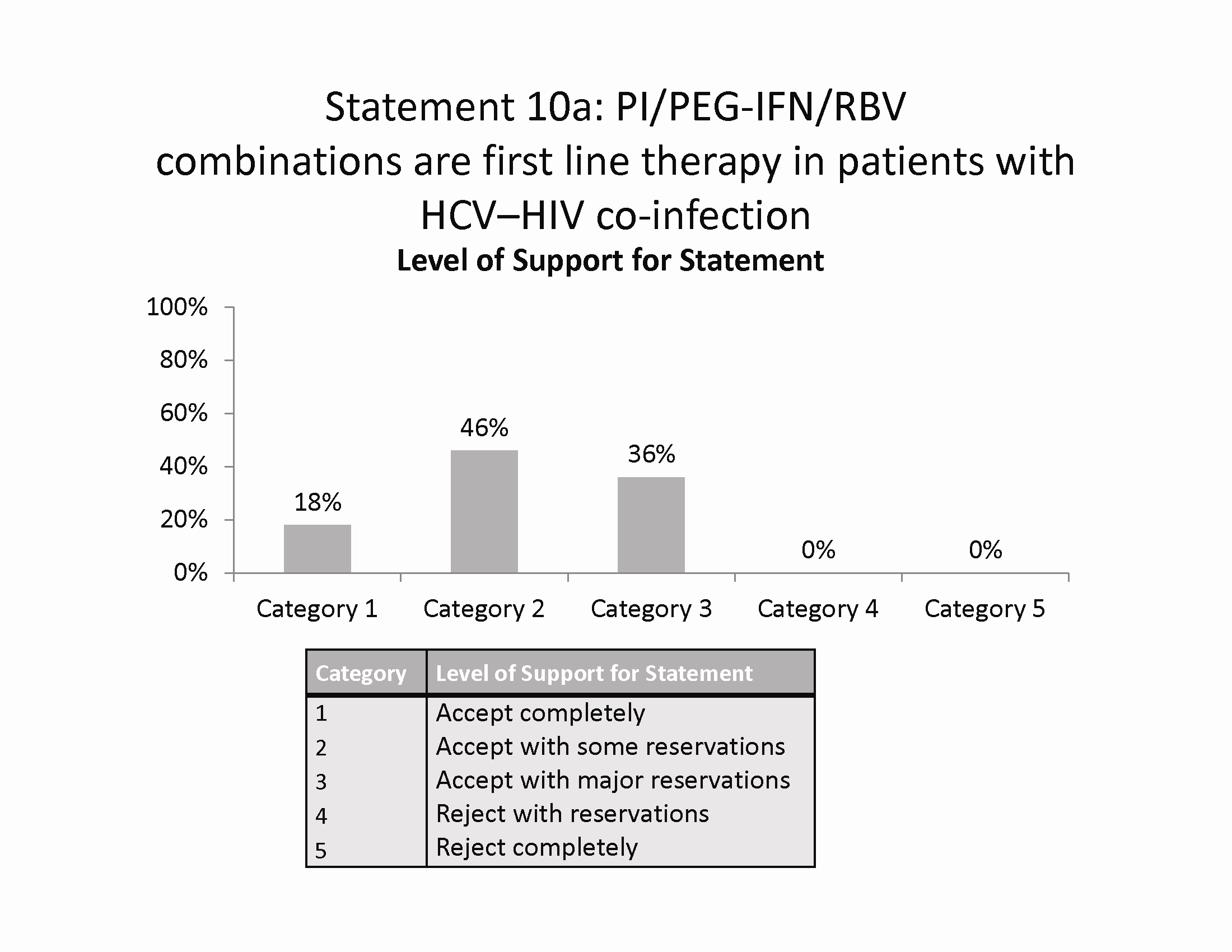** |
| **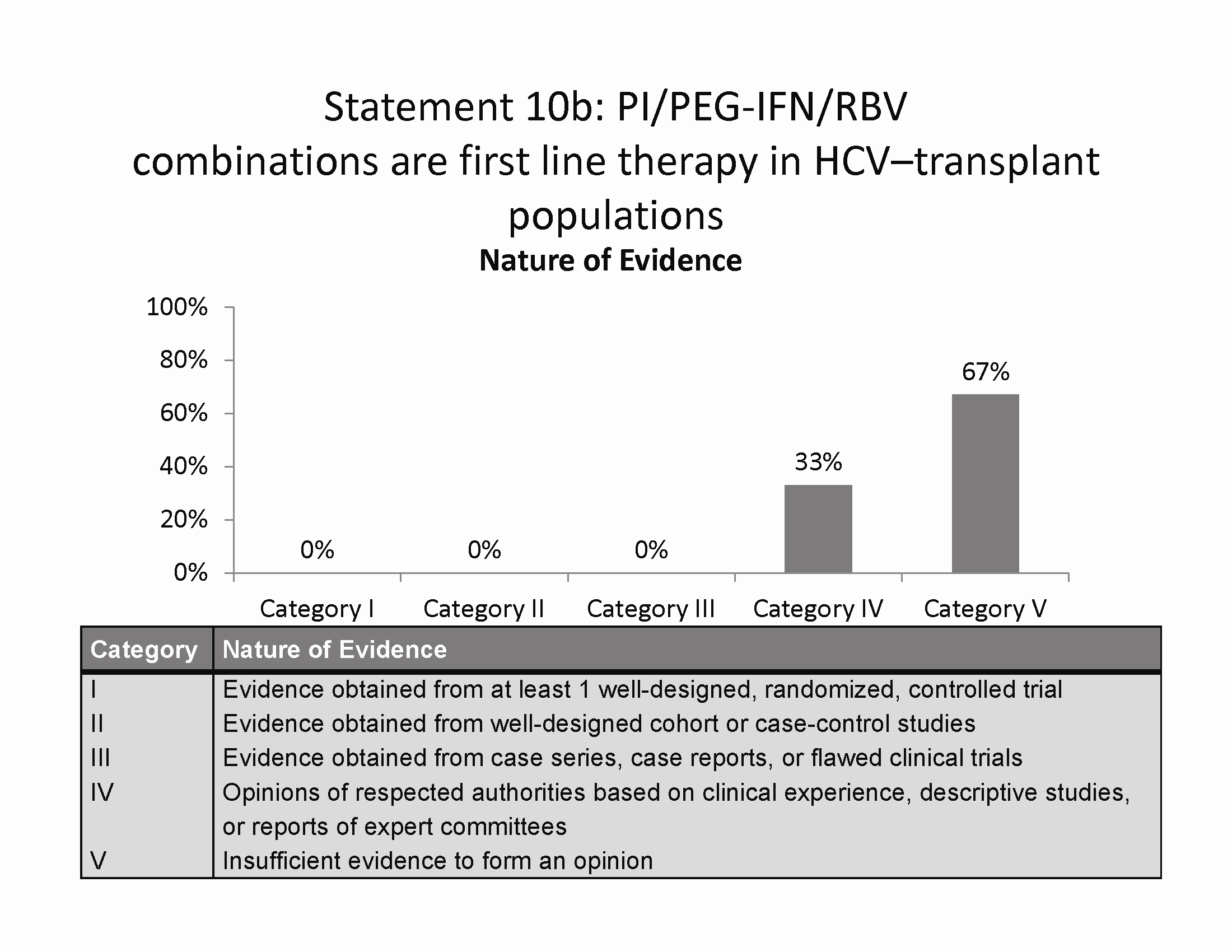** | **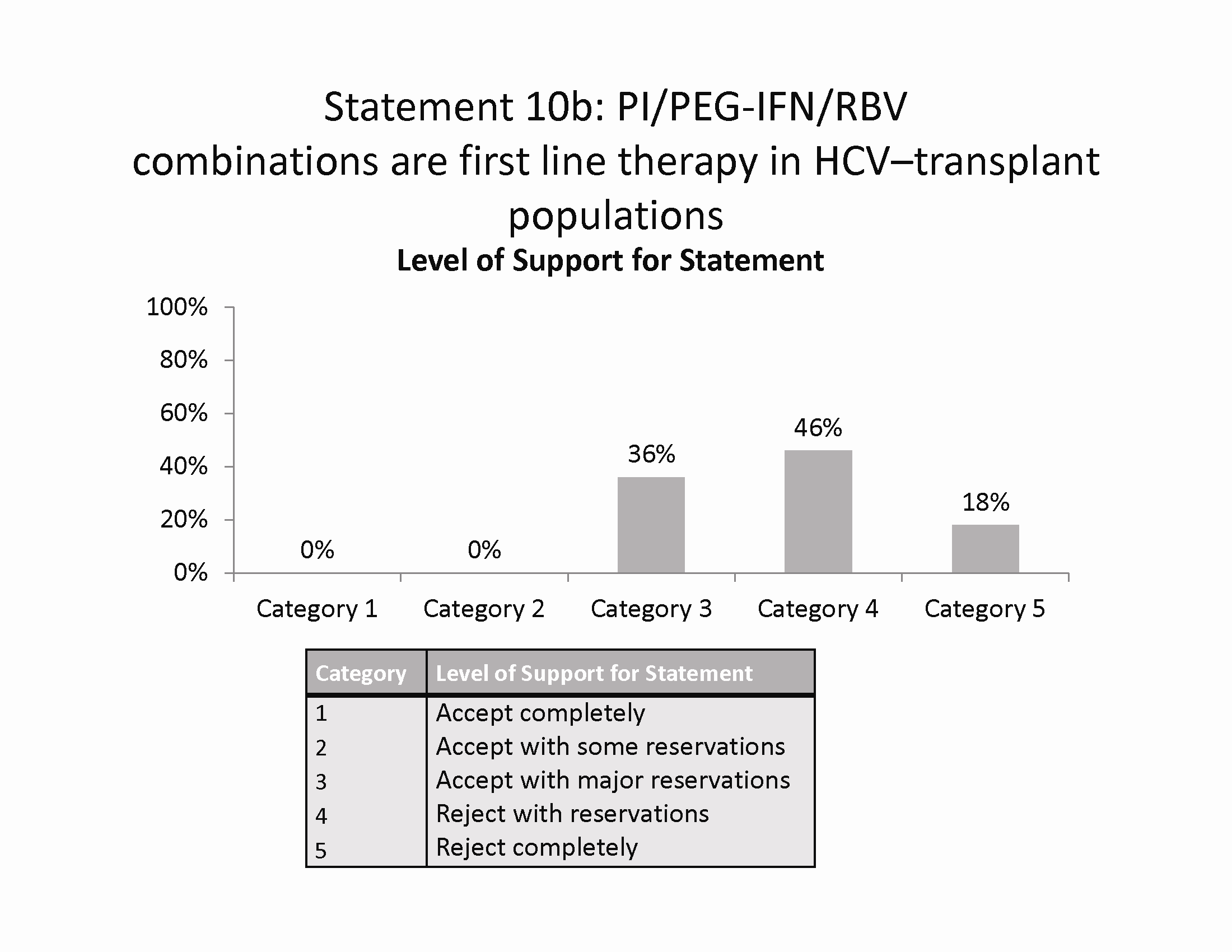** |

Supplement: Supplementary file 1 — The purpose of this Council was to critically review the existing body of literature and analyze it in the context of contemporary practice and optimal therapeutic outcomes. Each panel member focused on the existing evidence pertaining to a specific controversy in the management of patients with HCV, and the Council as a whole evaluated and voted on the nature of the existing evidence for each statement. The Council members' evaluations of the evidence with level of support for each statement are shown in the supplemental materials. [file 138302.f1.doc]
